# Molecular and serological detection of bovine babesiosis in Indonesia

**DOI:** 10.1186/s13071-017-2502-0

**Published:** 2017-11-06

**Authors:** Azirwan Guswanto, Puttik Allamanda, Euis Siti Mariamah, Sodirun Sodirun, Putut Eko Wibowo, Liliek Indrayani, Rudi Harso Nugroho, I Ketut Wirata, Nur Jannah, Lepsi Putri Dias, Hadi Purnama Wirawan, Rochmadi Yanto, Bumduuren Tuvshintulga, Thillaiampalam Sivakumar, Naoaki Yokoyama, Ikuo Igarashi

**Affiliations:** 1 National Research Center for Protozoan Diseases, Obihiro University of Agriculture and Veterinary Medicine, Nishi 2-13 Inada-cho, Obihiro, 080-8555 Japan; 2 Balai Veteriner Subang (DIC Subang), Jl. Terusan Garuda 33/11 Blok Werasari Dangdeur, Subang, Jawa Barat 41212 Indonesia; 3 Balai Veteriner Bukittinggi (DIC Bukittinggi), Jl. Raya Bukittinggi-, Payakumbuh, Tabek Panjang, Baso, Kabupaten Agam, Sumatera Barat 26192 Indonesia; 4 Balai Besar Veteriner Denpasar (DIC Denpasar), Jl. Raya Sesetan No. 266, Sesetan, Denpasar, Sel., Kota Denpasar, Bali 80223 Indonesia; 5 Balai Veteriner Banjarbaru (DIC Banjarbaru), Jln. Ambulung 24, Loktabat Selatan, Banjarbaru, Kalimantan Selatan 70712 Indonesia; 6 Balai Veteriner Medan (DIC Medan), Jl. Gatot Subroto No. 255-A, Lalang, Medan, Sunggal, Kota Medan, Sumatera Utara 20123 Indonesia; 7 Balai Besar Veteriner Maros (DIC Maros), Jl. Dr. Sam Ratulangi, Allepolea, Lau, Kabupaten Maros, Sulawesi Selatan 90512 Indonesia; 8 Balai Besar Veteriner Wates (DIC Wates), Jl. Raya Yogya-Wates Km. 27, TP 18, Giri Peni, Wates, Kabupaten Kulon Progo, Daerah Istimewa Yogyakarta 55602 Indonesia

**Keywords:** Bovine babesiosis, Serological detection, Molecular detection, Indonesia

## Abstract

**Background:**

Bovine babesiosis, mainly caused by *Babesia bovis* and *B. bigemina*, is a huge threat to the livestock industry. In Indonesia, the current distribution of the disease is unknown due to a lack of scientific study.

**Methods:**

In the present study, 487 blood samples were collected from cattle with different breeding and age groups in a broad geographical area across the archipelago. The presence of antibodies and current infections of *B. bovis* and *B. bigemina* were determined using enzyme-linked immunosorbent assay (ELISA), immunochromatographic test (ICT), and nested PCR (nPCR) targeting *B. bovis SBP-4* and *B. bigemina RAP-1a* genes. Sequence analysis was performed to the amplicon of *B. bovis SBP-4*, *B. bigemina RAP-1a*, and internal transcribed spacer (ITS) region of ribosomal RNA of both *Babesia* species.

**Results:**

In total, *B. bovis* positives were detected by ELISA, single-ICT, dual-ICT and nPCR in 340 (69.8%), 317 (65.1%), 307 (63.0%) and 247 (50.7%) samples, respectively. For *B. bigemina*, the positive samples were detected in 134 (27.5%), 130 (26.7%), 127 (26.1%) and 93 (19.1%), respectively. Furthermore, mixed infections were found in 125 (25.7%), 113 (23.2%), 109 (22.4%) and 52 (10.7%) samples, respectively, which occurred only by chance and were not influenced by additional factors. The obtained nucleotide sequences of *B. bovis SBP-4* and *B. bigemina RAP-1a* genes showed a high homology with other isolates from different countries. Further nucleotide sequence analysis using ITS region showed a great genetic diversity of *B. bovis* isolates among sampling locations; a lower diversity was found in *B. bigemina* ITS isolates.

**Conclusions:**

These data revealed the current distribution of *B. bovis* and *B. bigemina* infection in cattle in Indonesia. The rate of infection varied among sampling locations, cattle breeds and age groups. Furthermore, *B. bovis* ITS isolates from Indonesia were found to be more genetically diverse than *B. bigemina* ITS isolates. The data presented in this study are necessary to develop an effective strategy for controlling the disease in the country.

**Electronic supplementary material:**

The online version of this article (10.1186/s13071-017-2502-0) contains supplementary material, which is available to authorized users.

## Background

Bovine babesiosis is a huge threat to the livestock industry as it is associated with direct economic losses like loss of body weight and milk productions and death of animals, and indirect costs of prevention and treatment. Common *Babesia* species that infect cattle are *B. bovis*, *B. bigemina* and *B. divergens*. Tick vectors such as *Rhipicephalus microplus*, *R. annulatus* and *R. geigyi* can transmit *B. bovis* and *B. bigemina*, while *R. decoloratus* and *R. evertsi* can only transmit *B. bigemina*. *Babesia divergens* is usually transmitted by *Ixodes ricinus*. The distribution of tick vectors in many parts of the world correspond well with the presence of the parasite [1, 2]. During infected tick blood-feeding on cattle, sporozoites enter the host’s blood circulation and invade the red blood cells (RBCs), where they undergo asexual replication. The developmental stage in RBCs involves a few morphological changes, from round trophozoites to binary fission trophozoites and then merozoites. Mature merozoites rupture the cells and subsequently infect new RBCs. Ticks ingest some merozoites during blood-feeding, and sexual replication is initiated in the tick midgut. The parasites are transmitted transovarially to the tick eggs to continue the life-cycle [3, 4].

*Babesia* parasites create and stabilize their intracellular environment to make it suitable during their life-cycle. It involves the releasing of numerous molecules by apical complex of the parasite in all stage of asexual replication, including cell invasion, intracellular developments, and egress from the cell. Exploitation of released molecules, produced by apical complex of the parasite, has been shown to be an effective tool for the development of diagnostic methods and as the drug target in chemotherapeutic development [5]. The spherical body protein (SBP-4) of *B. bovis* is found in the spherical bodies, a component of apical complex. The protein is characterized by its abundance in the cytoplasm in the late stage of intracellular infection and released into blood circulation. Therefore, it has a greater possibility of reacting with the host immune systems during rupture of infected cells and has potential as serological diagnostic target [6]. The *B. bovis SBP-4* gene is conserved among isolates from different geographical areas and has a low homology with other apicomplexan parasites, indicating the suitability of the gene as a specific target for molecular diagnosis [7, 8]. Correspondingly, the utilization of *B. bovis SBP-4*-based serological and molecular diagnostic methods has been applied in several studies [9–12]. The rhoptry-associated protein 1 (RAP-1) is a conserved gene family in many *Babesia* species and plays an important role during parasite invasion [13, 14]. An enzyme-linked immunosorbent assay (ELISA) using this gene has been developed to detect antibodies of *B. bovis* and *B. bigemina*; however, the cross-reactivity was apparently high [15]. A previous study determined the high specificity of the truncated C-terminal RAP-1 for the serological detection of *B. bigemina* [16]. For molecular detection, *RAP-1a* is a highly conserved gene among *B. bigemina* isolates that has been utilized in several studies [9–11]. Above all, the application of specific and sensitive diagnostic methods is necessary to accurately determine the presence of *Babesia* parasites during routine surveillance.

In Indonesia, bovine babesiosis was first reported in 1896; the disease was later found to be endemic in the country [17]. In 1993, a study employing ELISA for the detection of *B. bovis* antibodies in cattle serum samples showed prevalences as high as 96% in the islands of Sumatera, Kalimantan, Sulawesi, Sumba and Timor [18]. The country also imports live cattle from several countries to meet domestic demands. Based on the microscopic observation of blood smears, one study reported that the average prevalence of babesiosis in cattle imported from Australia was 10.5% [19]. A recent study using a similar method showed a higher prevalence of babesiosis at 42.9% and a moderate rate of mixed infection of *Anaplasma* spp. and *Theileria* spp. [20]. The imported cattle did not show any clinical symptoms upon arrival. However, they could be the source of the subsequent infection for other cattle [20]. Indonesian cattle are not vaccinated against babesiosis, and the nation-wide surveillance program conducted by Veterinary Diagnostic Centers is based on microscopic observation. Therefore, studies to determine the distribution and genetic characterization of the *Babesia* parasites in the country are necessary to provide critical data for the development of effective measures for controlling the disease.

The main purpose of this study was to determine the distribution of bovine babesiosis in a wide geographical area across Indonesia. It also aimed to evaluate the potency of diagnostic methods, such as indirect ELISA (iELISA), immunochromatographic test (ICT) and nested PCR (nPCR), to be applied in field surveillance in the country. The final objective was to characterize the genetic diversity of Indonesian isolates based on the *SBP-4* gene of *B. bovis*, the *RAP-1a* gene of *B. bigemina*, and internal transcribed spacer (ITS) region of rRNA of *B. bovis* and *B. bigemina*.

## Methods

### Parasites and the preparation of recombinant protein *B. bovis* SBP-4 and *B. bigemina* RAP-1/CT17

*Babesia bovis* (Texas strain) and *B. bigemina* (Argentina strain) were cultured continuously using a microaerophilic culture system [21, 22]. The cultures were used to synthesize cDNA for the expression of *B. bovis* spherical body protein 4 (SBP-4) and *B. bigemina* C-terminal rhoptry-associated protein (RAP-1/CT17) in accordance with the method described previously [23, 24]. After measuring the concentration, the recombinant protein was injected into mice to produce polyclonal antibodies, and the remaining proteins were stored at -30 °C until the ICT strips were prepared and ELISA examinations were performed. Genomic DNA for positive control in PCR amplification was also obtained from the cultures.

### Study areas and blood sample collections

**Fig. 1:**
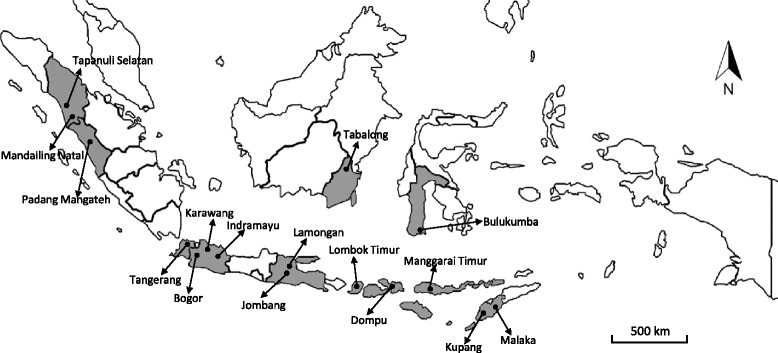
Geographical distribution of the sampling locations. A total of 487 blood samples were collected from clinically healthy cattle from sixteen locations across the Indonesian archipelago: Mandailing Natal (*n* = 32), Tapanuli Selatan (*n* = 28), Padang Mangateh (*n* = 60), Tangerang Regency (*n* = 18), Bogor Regency (*n* = 16), Karawang (*n* = 21), Indramayu (*n* = 10), Lamongan (*n* = 40), Jombang (*n* = 40), Tabalong (*n* = 60), Bulukumba (*n* = 74), Dompu (*n* = 17), Lombok Timur (*n* = 16), Kupang (*n* = 19), Manggarai Timur (*n* = 19) and Malaka (*n* = 17)

A total of 487 blood samples were randomly collected and divided into tubes, with and without anticoagulant, from clinically healthy cattle. Samples were collected from March to April 2016 in sixteen locations in Indonesia: Mandailing Natal (*n* = 32), Tapanuli Selatan (*n* = 28), Padang Mangateh (*n* = 60), Tangerang Regency (*n* = 18), Bogor Regency (*n* = 16), Karawang (*n* = 21), Indramayu (*n* = 10), Lamongan (*n* = 40), Jombang (*n* = 40), Tabalong (*n* = 60), Bulukumba (*n* = 74), Dompu (*n* = 17), Lombok Timur (*n* = 16), Kupang (*n* = 19), Manggarai Timur (*n* = 19) and Malaka (*n* = 17), as shown in Fig. 1. The breeds of cattle included Bali cattle (*n* = 207), Filial Ongole (*n* = 99), Brahman crossed (*n* = 88), Pesisir cattle (*n* = 60) and Taurine cattle (*n* = 33). The cattle were divided into three age groups, young (< 2 years), adult (2–4 years), and old (> 4 years). Serum samples were aliquoted into microcentrifuge tubes and transported to the laboratory with ice packs and stored at -30 °C before serological detection. Genomic DNA was purified from 200 μl of each blood sample using the QIAamp® DNA Blood Mini Kit (Qiagen, Hilden, Germany) in accordance with the manufacturer’s instructions. The DNA samples were stored at -30 °C until they were molecularly examined.

### Serological assays for the detection of antibody responses against *B. bovis* and *B. bigemina*

Three types of ICT strips were prepared, *bov*ICT, *big*ICT and dual-ICT. The ICTs were based on the recombinant proteins *B. bovis* SBP-4, *B. bigemina* RAP-1/CT17, and a combination of both proteins as detection antigens [24, 25]. ICT examinations were performed twice on each serum sample. A 20 μl serum sample was diluted with an equal amount of phosphate buffer saline (PBS) and applied to the sample pad of the ICT strip. The sample was determined to be seropositive if the clear band appeared in both the control and the test line after 15 min. For the iELISA, the recombinant proteins of *B. bovis* SBP-4 and *B. bigemina* RAP-1/CT17 were also used as detection antigens (*bov*ELISA and *big*ELISA). The sensitivity, specificity and cut-off value had been determined in a previous study [26]. iELISA, performed according to the protocol described elsewhere, was performed on all serum samples in duplicate [16].

### PCR screening for the detection of *B. bovis SBP-4* and *B. bigemina RAP-1a* genes

PCR assays targeted the *B. bovis SBP-4* and *B. bigemina RAP-1a* genes. The sets of primers, for both PCR and nPCR, were designed in accordance with the previously published work, as shown in Table 1 [26, 27]. Amplification was carried out using 10 μl of the reaction mixture, which contained 1 μl of each sample or control as a DNA template, 1 μl of 10× Ex Taq buffer (Takara Bio, Shiga, Japan), 1 μl of 8 mM dNTPs, 1 μl of 10 μM forward and reverse primers, and 0.1 U of Ex Taq polymerase (Takara Bio, Shiga, Japan), and brought to the total volume with distilled water. The PCR conditions for detecting *B. bovis* are as follows: the initial denaturation was at 94 °C for 1 min, followed by 35 cycles of denaturation at 94 °C for 30 s, annealing at 65 °C for 1 min, extension at 72 °C for 1 min, and a final extension at 72 °C for 7 min. The conditions for nPCR were similar except for the annealing temperature, which was 53.5 °C. For *B. bigemina*, the initial denaturation was at 95 °C for 5 min, followed by 35 cycles of denaturation at 94 °C for 1 min, annealing at 55 °C for 1 min, extension at 72 °C for 1 min, and a final extension at 72 °C for 10 min. Similar conditions were applied in nPCR for *B. bigemina* detection. The reaction mixture of the nPCR included the amplicon from the first PCR as a DNA template and the amplified fragments were analyzed using 1.5% agarose gel. The gel was subsequently stained with ethidium bromide and visualized under UV illumination.

**Table 1:** Target genes and primers used in this study [26, 27]

Species	Target gene	Method	Primer name	Oligonucleotide sequence (5′–3′)	Product size (bp)
*B. bovis*	*SBP-4*	PCR	bov-SBP-4-F	AGTTGTTGGAGGAGGCTAAT	907
			bov-SBP-4-R	TCCTTCTCGGCGTCCTTTTC	
		nPCR	bov-SBP-4-nF	GAAATCCCTGTTCCAGAG	503
			bov-SBP-4-nR	TCGTTGATAACACTGCAA	
*B. bigemina*	*RAP-1a*	PCR	big-RAP-1a-F	GAGTCTGCCAAATCCTTAC	879
			big-RAP-1a-R	TCCTCTACAGCTGCTTCG	
		nPCR	big-RAP-1a-nF	AGCTTGCTTTCACAACTCGCC	412
			big-RAP-1a-nR	TTGGTGCTTTGACCGACGACAT	
*B. bovis*	ITS region	PCR	bov-ITS-F	CGTCCCTGCCCTTTGTA	815
			bov-ITS-R	TATTTTCTTTTCTGCCGCTT	
		nPCR	bov-ITS-nF	CACCACCAGTGGAAGCAC	545
			bov-ITS-nR	TTGTGCCCCATGGACACT	
*B. bigemina*	ITS region	PCR	big-ITS-F	CGTCCCTGCCCTTTGTA	1041
			big-ITS-R	TATTTTCTTTTCTGCCGCTT	
		nPCR	big-ITS-nF	AGTGGTCGGGACTCGTC	495
			big-ITS-nR	AGTACCGCGTGCGAGCAG	

### Genetic characterization of *B. bovis SBP-4*, *B. bigemina RAP-1a*, and ITS region of *B. bovis* and *B. bigemina*

Positive DNA samples in the PCR screening were selected randomly for the amplification of ITS region of *B. bovis* (*n* = 9) and *B. bigemina* (*n* = 8). The sets of primers for the amplification of ITS1–5.8S rRNA gene-ITS2 regions from *B. bovis* and *B. bigemina* are shown in the Table 1 [27]. The reaction mixture contained 1 μl of each sample, 2 μl of 5× SuperFi buffer (Invitrogen, Carlsbad, CA, USA), 1 μl of 8 mM dNTPs, 1 μl of 10 μM forward and reverse primers, and 0.2 U of Platinum SuperFi DNA polymerase (Invitrogen, Carlsbad, CA, USA), and brought to the total volume of 10 μl with distilled water. The PCR conditions for the amplification of *B. bovis* and *B. bigemina* ITS regions were as follows. The initial denaturation was at 98 °C for 30 s, followed by 35 cycles of denaturation at 98 °C for 10 s, annealing at 60.2 °C for 10 s, extension at 72 °C for 30 s, and a final extension at 72 °C for 5 min. For the nPCR, similar conditions were used except the annealing temperature was 64.9 °C and 63.3 °C for *B. bovis* ITS and *B. bigemina* ITS, respectively. The amplified fragments were analyzed using 1.5% agarose gel.

Gel purifications were conducted to the amplicon of *B. bovis* ITS, *B*. *bigemina* ITS regions, *B. bovis SBP-4* (*n* = 16), and *B. bigemina RAP-1a* (*n* = 13) using NucleoSpin® Gel and PCR Clean-up (Macherey-Nagel, Düren, Germany). Subsequently, the purified fragments were cloned into the pCR™ 2.1-TOPO® TA vector (Invitrogen, Carlsbad, CA, USA) and transformed into *Escherichia coli* TOP 10 competent cells (Invitrogen, Carlsbad, CA, USA). Two single colonies from each *E. coli*-transformed clone were selected and amplified with pCR™ 2.1-TOPO® TA vector primers. Sequence analysis was performed in both forward and reverse directions using a BigDye® Terminator v3.1 Cycle Sequencing Kit and an ABI PRISM 3130XL Genetic Analyzer (Applied Biosystems, Foster City, CA, USA).

### Bioinformatics analysis

All obtained sequences were analyzed using the Basic Local Alignment Search Tool for nucleotides (BLASTn) [28] and pairwise sequence alignment by EMBOSS Needle [29] to find the homologs in the database. The typical nucleotide sequence from each isolate was then processed for further multiple sequence alignments using Clustal Omega [29]. Finally, the aligned sequences were analyzed by the maximum likelihood estimation method to construct a phylogenetic tree using MEGA 7.0.21 software [30].

### Statistical analysis

The strength of agreement between ELISA, *bov*ICT, *big*ICT, and dual-ICT was evaluated using Kappa statistics [31]. Significant differences (*P* < 0.05) of *B. bovis* and *B. bigemina* detections between sampling locations, cattle breeds, age of cattle and the expected conditional probability of mixed infections were analyzed using a Chi-square test utilizing EpiTools epidemiological calculators [32]. Furthermore, DNA sequence polymorphism analysis was conducted to determine the number of haplotypes (*h*), nucleotide diversity (*π*), and average number of nucleotide differences (*k*) using DnaSP version 5 [33]**.**

## Results

### Serological detection of *B. bovis* and *B. bigemina*

The serological detections in this study were based on ELISA and ICT systems to detect specific antibodies against *B. bovis* and *B. bigemina* in cattle. ELISA and ICT examinations revealed a high positive rate of antibody response against *B. bovis* (Table 2). Among all sampling locations, only two sites, Lamongan and Jombang, had a positive rate as low as 10%. Other locations reached 100% positive rates, such as in Manggarai Timur and Malaka. In total, 340 (69.8%), 317 (65.1%), and 307 (63.0%) serum samples were positive for *B. bovis* antibody when using *bov*ELISA, *bov*ICT and dual-ICT, respectively. The positive rates of *B. bigemina* infections were lower than those of *B. bovis*. We found no positive case in Jombang and Lamongan by *big*ELISA and *big*ICT; however, 1 (2.5%) serum sample from Lamongan was seropositive by dual-ICT. From the total samples, *big*ELISA, *big*ICT and dual-ICT detected positive antibody responses to *B. bigemina* in 134 (27.5%), 130 (26.7%), and 127 (26.15%) serum samples, respectively. Mixed infections by both parasites were detected in 125 (25.7%), 113 (23.2%) and 109 (22.4%) samples, respectively. Analysis of expected conditional probability of mixed infection (Additional file 1: Tables S1-S3) showed that the observed number of cattle with both *B. bovis* and *B. bigemina* infections were not significantly different, with the expected number in all sampling locations. The events only occurred by chance and were not influenced by additional factors. Furthermore, agreement between ELISA and ICT was analyzed using Kappa statistics (Table 3). For *B. bovis*, the Kappa values between *bov*ICT, dual-ICT, and *bov*ELISA ranged between 0.743–0.927, which indicated satisfactory agreement between the methods. Similar results were also determined for *B. bigemina*, with the Kappa values between *big*ICT, dual-ICT and *big*ELISA ranging between 0.748–0.888.

**Table 2:** ELISA, ICTs and nPCR results of *Babesia bovis* and *Babesia bigemina* in all sampling locations

Sampling location	No. of samples	No. positive (%)											
		*B. bovis*				*B. bigemina*				Mixed infection			
		*bov*ELISA	*bov*ICT	dual-ICT	nPCR	*big*ELISA	*big*ICT	dual-ICT	nPCR	*bov/big*-ELISA	*bov/big*-ICT	dual-ICT	nPCR
Karawang	21	18 (85.7)	17 (81.0)	17 (81.0)	7 (33.3)	9 (42.9)	8 (38.1)	8 (38.1)	2 (9.5)	9 (42.9)	8 (38.1)	8 (38.1)	1 (4.8)
Tangerang	18	16 (88.9)	13 (72.2)	14 (77.8)	11 (61.1)	15 (83.3)	14 (77.8)	12 (66.7)	1 (5.6)	14 (77.8)	11 (61.1)	10 (55.6)	0 (0.0)
Bogor	16	13 (81.3)	10 (62.5)	11 (68.8)	7 (43.8)	13 (81.3)	10 (62.5)	10 (62.5)	2 (12.5)	10 (62.5)	6 (37.5)	7 (43.8)	2 (12.5)
Indramayu	10	4 (40.0)	4 (40.0)	4 (40.0)	2 (20.0)	4 (40.0)	4 (40.0)	3 (30.0)	2 (20.0)	2 (20.0)	2 (20.0)	2 (20.0)	0 (0.0)
Padang Mangateh	60	59 (98.3)	51 (85.0)	51 (85.0)	13 (21.7)	26 (43.3)	25 (41.7)	25 (41.7)	28 (46.7)	26 (43.3)	21 (35.0)	21 (35.0)	6 (10.0)
Dompu	17	16 (94.1)	15 (88.2)	15 (88.2)	3 (17.6)	10 (58.8)	10 (58.8)	11 (64.7)	3 (17.6)	10 (58.8)	9 (52.9)	10 (58.8)	1 (5.9)
Lombok Timur	16	9 (56.3)	8 (50.0)	8 (50.0)	14 (87.5)	1 (6.3)	2 (12.5)	2 (12.5)	6 (37.5)	1 (6.3)	2 (12.5)	2 (12.5)	5 (31.3)
Kupang	19	17 (89.5)	18 (94.7)	17 (89.5)	17 (89.5)	2 (10.5)	3 (15.8)	3 (15.8)	5 (26.3)	2 (10.5)	3(15.8)	3 (15.8)	4 (21.1)
Manggarai Timur	19	19 (100)	19 (100)	19 (100)	18 (94.7)	7 (36.8)	7 (36.8)	7 (36.8)	7 (36.8)	7 (36.8)	7 (36.8)	7 (36.8)	7 (36.8)
Malaka	17	17 (100)	17 (100)	17 (100)	14 (82.4)	7 (41.2)	8 (47.1)	8 (47.1)	6 (35.3)	7 (41.2)	8 (47.1)	8 (47.1)	5 (29.4)
Tabalong	60	50 (83.3)	47 (78.3)	40 (66.7)	30 (50.0)	14 (23.3)	11 (18.3)	8 (13.3)	6 (10.0)	13 (21.7)	10 (16.7)	6 (10.0)	5 (8.3)
Mandailing Natal	32	29 (90.6)	27 (84.4)	27 (84.4)	14 (43.8)	9 (28.1)	10 (31.3)	10 (31.3)	12 (37.5)	9 (28.1)	9 (28.1)	9 (28.1)	8 (25.0)
Tapanuli Selatan	28	27 (96.4)	24 (85.7)	23 (82.1)	14 (50.0)	9 (32.1)	11 (39.3)	11 (39.3)	5 (17.9)	8 (28.6)	10 (35.7)	9 (32.1)	3 (10.7)
Bulukumba	74	38 (51.4)	42 (56.8)	39 (52.7)	51 (68.9)	8 (10.8)	7 (9.5)	8 (10.8)	6 (8.1)	7 (9.5)	7 (9.5)	7 (9.5)	5 (6.8)
Lamongan	40	4 (10.0)	2 (5.0)	2 (5.0)	18 (45.0)	0	0	1 (2.5)	1 (2.5)	0	0	0	0 (0.0)
Jombang	40	4 (10.0)	3 (7.5)	3 (7.5)	14 (35.0)	0	0	0	1 (2.5)	0	0	0	0 (0.0)
Total	487	340 (69.8)	317 (65.1)	307 (63.0)	247 (50.7)	134 (27.5)	130 (26.7)	127 (26.1)	93 (19.1)	125 (25.7)	113 (23.2)	109 (22.4)	52 (10.7)

**Table 3:** Agreement between serological methods

Diagnostic methods	Kappa value	95% CI^a^	Agreement^b^
*bov*ICT and *bov*ELISA	0.744	0.688–0.800	Good
dual-ICT and *bov*ELISA	0.743	0.687–0.798	Good
*bov*ICT and dual-ICT	0.927	0.896–0.957	Very good
*big*ICT and *big*ELISA	0.841	0.793–0.889	Very good
dual-ICT and *big*ELISA	0.748	0.689–0.808	Good
*big*ICT and dual-ICT	0.888	0.847–0.929	Very good

^a^95% confidence interval

^b^Agreement was analyzed using kappa statistics and stated as poor (< 0.20), fair (0.21–0.40), moderate (0.41–0.60), good (0.61–0.80), or very good (0.81–1.00) [31]

### Molecular detection of *B. bovis* and *B. bigemina*

Nested PCR detection targeted the highly conserved regions of *B. bovis SBP-4* and *B. bigemina RAP-1a* genes. The negative controls consisted of genomic DNA from healthy cattle, which had been kept in our laboratory, and the elution buffer used during DNA extraction did not show any amplification in the first PCR or the nested PCR. The positive rate of *B. bovis* infection was more than 30% in most sampling locations, except in Indramayu, Padang Mangateh and Dompu (Table 2). In contrast, the infection rates of *B. bigemina* in most locations were lower than 30%, except in Padang Mangateh, Lombok Timur, Manggarai Timur, Malaka and Mandailing Natal. In total, *B. bovis* and *B. bigemina* were detected in 247 (50.7%) and 93 (19.1%) of cattle DNA samples, respectively. Mixed infections by both parasites were detected in 52 samples (10.7%). Similar with serological detections, analysis of expected conditional probability of mixed infection (Additional file 1: Table S4) showed that the event only occurred by chance and were not affected by other factors.

### Comparison of detections by ELISA, ICT, and nPCR

We also compared the results of the different methods used in this study (Table 4). The positive rate of ICT was lower than that of ELISA, but the correlation between methods was high (*χ* ^2^= 286.14, *df* = 1, *P* < 0.0001). A high correlation was also determined on the detection of *B. bigemina* by nPCR (*χ* ^2^= 7.22, *df* = 1, *P* = 0.01). On the other hand, there was no correlation between ELISA and nPCR in the detection of *B. bovis* (*χ* ^2^= 0.094, *df* = 1, *P* = 0.759). Both ELISA and nPCR detected positive *B. bovis* in 174 (35.7%) samples, while 166 (34.1%) samples were detected as positive by ELISA only, 73 (15.0%) samples tested positive by nPCR only, and 74 (15.2%) samples tested negative by both methods. For *B. bigemina*, only 36 (7.4%) samples were positive by both methods, while 98 (20.1%) samples were positive by ELISA only, 57 (11.7%) samples were positive by nPCR only, and 296 (60.8%) samples remained negative by both methods. Additionally, high titer antibodies in ELISA were more likely to be positive by nPCR. Some early infections of *B. bovis* were detected in Jombang, Lamongan, Bulukumba and Lombok Timur, as indicated by the higher nPCR positive rate than for those with ELISA. Furthermore, a higher nPCR positive rate of *B. bigemina* was observed in Padang Mangateh, Lombok Timur, Kupang, Mandailing Natal, Jombang and Lamongan.

**Table 4:** Comparison of the results summary of ELISA, ICT and nested PCR

Species	ELISA		*bov*ICT/*big*ICT			Dual-ICT			Nested PCR		
			(+)	(−)	*P*-value	(+)	(−)	*P*-value	(+)	(−)	*P-*value
*B. bovis*	(+)	340 (69.8)	303 (62.2)	37 (7.6)	< 0.0001	299 (61.4)	41 (8.4)	< 0.0001	174 (35.7)	166 (34.1)	0.759
	(−)	147 (30.2)	14 (2.9)	133 (27.3)		8 (1.6)	139 (28.5)		73 (15.0)	74 (15.2)	
	Total	487	317 (65.1)	170 (34.9)		307 (63.0)	180 (37.0)		247 (50.7)	240 (49.3)	
*B. bigemina*	(+)	134 (27.5)	121 (24.8)	13 (2.7)	< 0.0001	110 (22.6)	24 (4.9)	< 0.0001	36 (7.4)	98 (20.1)	0.010
	(−)	353 (72.5)	9 (1.8)	344 (70.6)		17 (3.5)	336 (69.0)		57 (11.7)	296 (60.8)	
	Total	487	130 (26.7)	357 (73.3)		127 (26.1)	360 (73.9)		93 (19.1)	394 (80.9)	

### Effect of breeds and age groups of cattle on *B. bovis* and *B. bigemina* infections

**Fig. 2:**
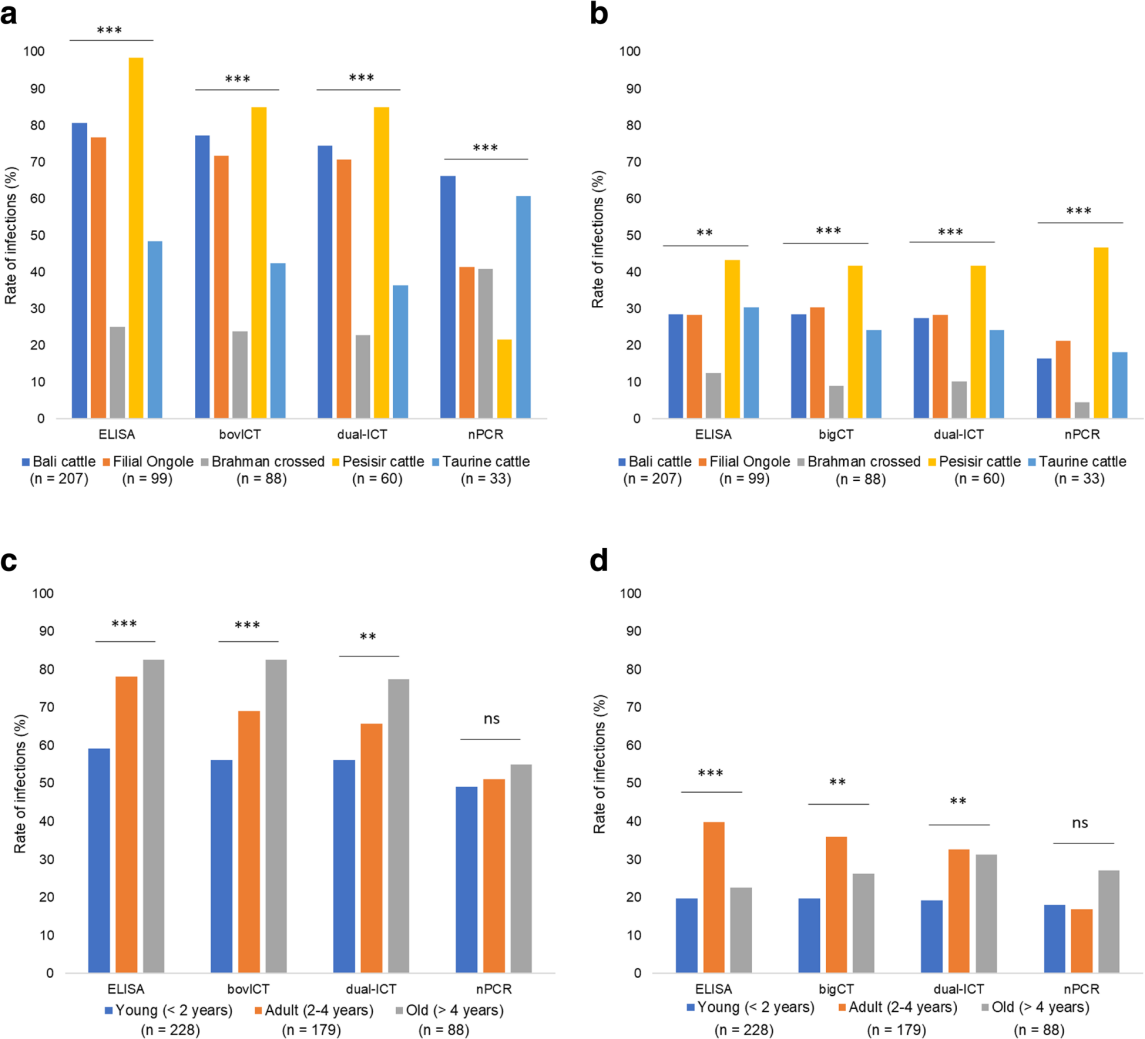
Comparison of positive rates between the breeds and age groups of cattle: (**a**) and (**b**) show the effect of breeds on the infection of *B. bovis* and *B. bigemina*, respectively; (**c**) and (**d**) show the effect of age groups on the infection of *B. bovis* and *B. bigemina*, respectively. The data were analyzed using Chi-square test (ns, *P* ≥ 0.05; *P < 0.05; **P < 0.01; ***P < 0.001)

The rates of *B. bovis* infection were significantly different among cattle breeds using ELISA (*χ* ^2^= 128.01, *df* = 4, *P* < 0.0001), *bov*ICT (*χ* ^2^= 99.24, *df* = 4, *P* < 0.0001), dual-ICT (*χ* ^2^= 97.83, *df* = 4, *P* < 0.0001), and nPCR (*χ* ^2^= 48.18, *df* = 4, *P* < 0.0001) as shown in Fig. 2a. Pesisir cattle exhibited the highest positive rate of *B. bovis* using ELISA (98.3%), *bov*ICT (85%) and dual-ICT (85%), but the lowest using nPCR (21.7%). Brahman crossed cattle showed the lowest positive rate of *B. bovis* infection among other breeds using ELISA (25%), *bov*ICT (23.9%) and dual-ICT (22.7%), but a higher rate was detected by nPCR (40.9%). The rates of *B. bigemina* infection were also significantly different between cattle breeds using ELISA (*χ* ^2^= 17.73, *df* = 4, *P* = 0.0014), *big*ICT (*χ* ^2^= 21.92, *df* = 4, *P* = 0.0002), dual-ICT (*χ* ^2^= 19.57, *df* = 4, *P* = 0.0006), and nPCR (*χ* ^2^= 42.84, *df* = 4, *P* < 0.0001) as shown in Fig. 2b. The highest positive rate was found in Pesisir cattle (43.3, 41.7, 41.7 and 46.7%) and the lowest was found in Brahman crossed cattle (12.5, 9.1, 10.2 and 4.5%) using ELISA, *big*ICT, dual-ICT, and nPCR, respectively. The age of cattle also affected the rates of *B. bovis* infection using ELISA (*χ* ^2^= 24.12, *df* = 2, *P* < 0.0001), *bov*ICT (*χ* ^2^= 20.0, *df* = 2, *P* < 0.0001) and dual-ICT (*χ* ^2^= 12.41, *df* = 2, *P* = 0.0002). However, the difference was not significant when using nPCR (*χ* ^2^= 0.829, *df* = 2, *P* = 0.661) as shown in Fig. 2c. Young cattle were likely to have less infection compared with older cattle. For *B. bigemina*, the age of cattle significantly affected the rate of infection using ELISA (*χ* ^2^= 21.56, *df* = 2, *P* < 0.0001), *big*ICT (*χ* ^2^= 13.43, *df* = 2, *P* = 0.0012), and dual-ICT (*χ* ^2^= 10.44, *df* = 2, *P* = 0.0054), as shown in Fig. 2d. Similar with *B. bovis*, no significant difference was observed in the detection using nPCR (*χ* ^2^= 4.47, *df* = 2, *P* = 0.107). Adult cattle aged 2–4 years have the highest infection rate of *B. bigemina* compared with young and old groups using ELISA, *big*ICT and dual-ICT, but the lowest using nPCR.

### Sequence analysis

All sequences obtained from each sample were aligned to acquire the representative sequence. Nucleotide BLAST analysis of the sequences was homologous with respective gene targets of *B. bovis SBP-4*, *B. bigemina RAP-1a* and *B. bovis* and *B. bigemina* ITS regions in the database. The results confirmed the specificity of our nPCR assay for both *Babesia* species. For *B. bovis SBP-4*, the identities among isolates in this study and database ranged between 96.0–100%. Thirteen isolates with a product size of 503 bp shared 99.0–100% similarity with isolates from Mongolia, Egypt, Texas, Brazil, Syria, Thailand and Mexico. Two isolates from Dompu and Malaka, with a product size of 512 bp, and an isolate from Mandailing Natal (503 bp) shared between 96.0–97.0% similarity with isolates from the African continent. The multiple sequence alignment of the *B. bovis SBP-4* gene sequences among 16 Indonesian isolates revealed 57 nucleotide substitutions, of which 31 affected the modifications of 23 amino acids (aa). Most of the aa modifications were identified in Malaka, Dompu and Mandailing Natal (Additional file 2: alignments A, B). The percentage identities among Indonesian *B. bovis SBP-4* isolates ranged between 93.5–100% (Additional file 3: Table S5). The phylogenetic tree constructed of *B. bovis SBP-4* gene sequences showed that 13 Indonesian isolates were among other isolates from across continents in clade 1 (Additional file 4: Figure S1). Clade 2 was formed by isolates from Dompu and Malaka, while isolates from Mandailing Natal joined the third clade, together with isolates from African countries.

Indonesian *B. bigemina RAP-1a* isolates consisted of two types. Type 1 included seven Indonesian isolates, mostly from the western part of Indonesia, and shared 99.0–100% identities with isolates from across continents including Asia, South America, and Africa. In type 2, six Indonesian isolates, mostly found in the Nusa Tenggara area, shared 98–99% identities with sequences from Australia. The multiple sequence alignment of Indonesian *B. bigemina RAP-1a* gene sequence showed 31 nucleotide substitutions, of which 26 substitutions affected the modifications of 24 aa (Additional file 2: alignments C, D). The percentage identities among Indonesian *B. bigemina RAP-1a* isolates ranged between 94.4–100% (Additional file 3: Table S6). The phylogenetic tree of *B. bigemina RAP-1a* formed three clades (Additional file 4: Figure S2). Clade 1 constituted seven Indonesian isolates (Lamongan, Karawang, Jombang, Bulukumba, Padang Mangateh, Mandailing Natal, and Bogor) together with isolates from Brazil, Thailand, Syria, Benin, Egypt, Puerto Rico, the Philippines, Uruguay, Kenya, Turkey and Argentina. Four Indonesian isolates (Tabalong, Tangerang, Lombok Timur and Manggarai Timur) were branched into clade 2, together with the isolates from Australia. The last clade was specifically formed by isolates from Kupang and Malaka.

**Fig. 3:**
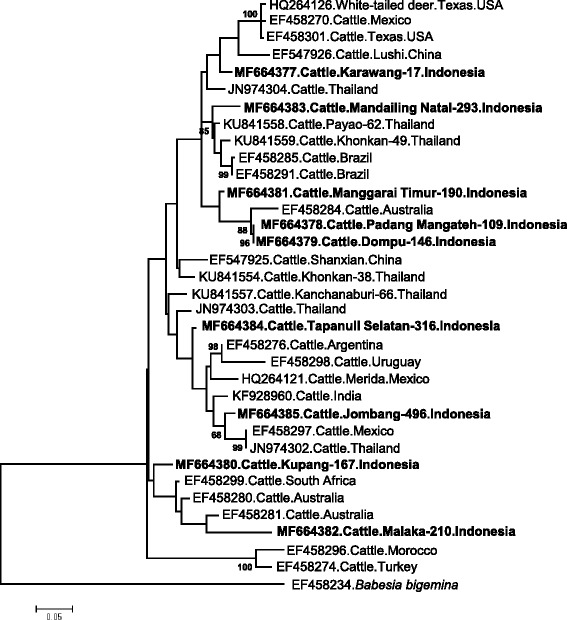
Phylogenetic tree based on *Babesia bovis* ITS1 partial sequence, 5.8S rRNA complete sequence, and ITS2 partial sequence data from Indonesian isolates and other sequences from GenBank. The maximum likelihood method based on the Kimura 2-parameter model with 1000 bootstrap replicates, available in MEGA ver.7, was used to determine the evolutionary history [30, 40]. All positions containing gaps and missing data were eliminated. Indonesian isolates are indicated by bold font

**Fig. 4:**
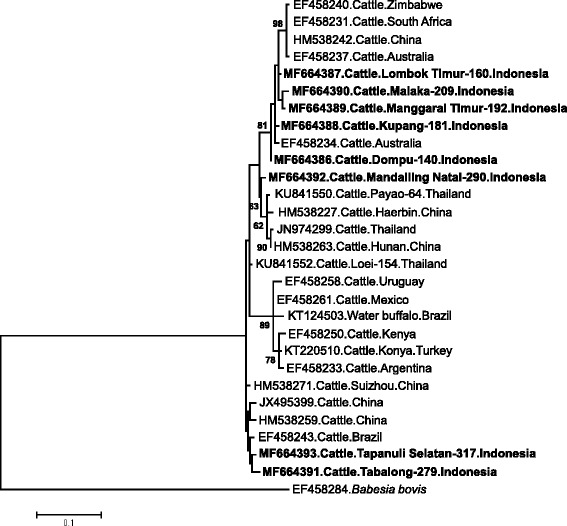
Phylogenetic tree based on *Babesia bigemina* ITS1 partial sequence and 5.8S rRNA partial sequence data from Indonesian isolates and other sequences from GenBank. The maximum likelihood method based on the Kimura 2-parameter model with 1000 bootstrap replicates, available in MEGA ver.7, was used to determine the evolutionary history [30, 40]. All positions containing gaps and missing data were eliminated. Indonesian isolates are indicated by bold font

Further characterizations were conducted to ITS regions of *B. bovis* and *B. bigemina*. The nucleotide sequences of Indonesian *B. bovis* ITS1–5.8S rRNA-ITS2 isolates in this study had a range of length between 487 and 508 bp. The sequences shared 91.0–96.0% sequence identity with isolates from Thailand, Australia, Brazil, South Africa and Mexico in the BLAST analysis. Among Indonesian isolates, the percent identities ranged between 81.4–99.6% (Additional file 2: alignment E; Additional file 3: Table S7). Phylogenetic analysis incorporating another 25 isolates from several countries revealed seven genotypes of Indonesian *B. bovis* ITS isolates (Fig. 3). For *B. bigemina*, the length of ITS1–5.8S rRNA nucleotide sequences ranged from 495 to 498 bp. Nucleotide BLAST analysis revealed the high identities of Indonesian *B. bigemina* ITS isolates with the isolates from South Africa, Australia, China, Thailand and Brazil. The percent identities between *B. bigemina* ITS sequences from Indonesia ranged from 93.5–98.8% (Additional file 2: alignment F; Additional file 3: Table S8). Unlike *B. bovis*, *B. bigemina* ITS isolates from Indonesia was less diverse in the phylogenetic analysis (Fig. 4).

Indonesian *B. bovis SBP-4*, *B. bigemina RAP-1a*, *B. bovis* ITS, and *B. bigemina* ITS isolates were further analyzed for the diversity of nucleotide sequences and nucleotide differences (Table 5). The highest nucleotide diversity (*π*) was determined in *B. bovis* ITS (0.079) with 35.889 of average number of nucleotide differences compared with *B. bigemina* ITS (*π* = 0.032 and *k* = 15.786), *B. bigemina RAP-1a* (*π* = 0.028 and *k* = 10.590) and *B. bovis SBP-4* (*π* = 0.018 and *k* = 9.142).

**Table 5:** Nucleotide polymorphism analysis of *B. bovis* and *B. bigemina* isolates from Indonesia

Target gene	*n*	No. of sites	*h*	*hd*	*π*	*k*
*B. bovis SBP-4*	16	512	12	0.942	0.018	9.142
*B. bigemina RAP-1a*	13	412	10	0.949	0.028	10.590
*B. bovis ITS* region	9	538	8	0.972	0.079	35.889
*B. bigemina* ITS region	8	500	8	1.000	0.032	15.786

*Abbreviations: N* number of sequences, *h* number of haplotypes, *hd* haplotype diversity, *π* nucleotide diversity, *k* average number of nucleotide differences

### The accession number of sequence nucleotides

Nucleotide sequences of *B. bovis SBP-4* (*n* = 16), *B. bigemina RAP-1a* (*n* = 13), *B. bovis* ITS1–5.8S rRNA-ITS2 (*n* = 9) and *B. bigemina* ITS1–5.8S rRNA (*n* = 8) were submitted to GenBank. The sequences can be retrieved with accession numbers KY484510–KY484534, KY562845–KY562848 and MF664377–MF664393.

## Discussion

The economic losses to the livestock industry due to bovine babesiosis are underestimated in Indonesia. A nationwide surveillance program that relies upon microscopic observation of Giemsa-stained blood smears leads to overlooking the infection. Usually, the prevalence of *Babesia* spp. detected by microscopy was lower than 5% in many locations in Indonesia. Under those circumstances, sporadic outbreaks typically occurred in some locations of the country and usually went unreported. In Indonesia, the predominant *Babesia* species that infect cattle are *B. bovis* and *B. bigemina*. It is assumed that they are both are transmitted by the common tick vector *R. microplus*, which displays a widespread distribution in the country [34]. A tick eradication program is absent in the country; however, farmers sometimes use acaricide to control tick infestations in their cattle. Given that, a program to control the disease should be supported by a more sensitive method of providing reliable data so as to avoid financial losses by farmers.

In this study, we reported the distribution of bovine babesiosis in a wide geographical area of Indonesia. Employing serological methods such as ELISA and ICT strips and molecular methods such as nPCR, we determined the rate of *B. bovis* and *B. bigemina* infections in cattle blood samples collected from 16 locations across the country. We chose recombinant proteins of *B. bovis* SBP-4 and *B. bigemina* RAP-1/CT17 as detection antigens in our ELISA and ICT methods. Both proteins showed a high sensitivity and specificity in the detections of specific antibodies against *B. bovis* and *B. bigemina* [6, 24, 25]. For nPCR, we chose *B. bovis SBP-4* and *B. bigemina RAP-1a* genes as detection targets. Each gene is specific for and conserved in each *Babesia* species, and there is low homology between them [7, 16, 23]. A combination of these diagnostic methods will allow the elucidation of the presence of a chronic or acute infection in the studied samples. In the early stage of *Babesia* infections, parasites appear in the red blood cells of host and can be detected by microscopy or DNA detection by PCR. However, antibodies against the parasites take days or weeks to produce and last for months during the chronic phase even after the parasites disappeared from red blood cells. In this phase, serological method such as ELISA or ICT would be a more useful diagnostic method [4].

The rates of *B. bovis* infection differed significantly among sampling locations. Serological detections showed a high positive rate of *B. bovis* infection in most sampling locations, except two locations, Lamongan and Jombang, which exhibited a low rate of positive antibodies. In contrast, molecular detection revealed a higher positive rate of *B. bovis* infection in these two locations. The predominant cattle breed in our samples from Lamongan and Jombang was Brahman crossed. This breed is known to be more resistant to the effect of *B. bovis* infection, which reduces the level of immunity in population. If the herd are exposed by high number of infected ticks, the possibility of new introduction of *B. bovis* is high [35]. The areas should avoid introducing new cattle from the endemic area since the herds in these locations are susceptible to infection. Other locations with high positive rate of *B. bovis* in both diagnostic methods might develop the enzootic stability because the cattle were asymptomatic during sample collections. The risk of outbreak is high if the susceptible animals are introduced into those locations and farmers and veterinary service organizations should be aware of this condition.

*Babesia bigemina* had a lower infection rate if compare with *B. bovis* in Indonesian cattle. At all sampling locations, the seropositivity of *B. bovis* was higher than that of *B. bigemina* by ELISA and ICTs, except in Bogor and Indramayu. These two locations exhibited an equal positive rate of antibodies for both *Babesia* species. Molecular examinations of DNA samples also revealed higher positive rates of *B. bovis* than of *B. bigemina*, except in samples from Indramayu, Dompu and Padang Mangateh. In Indramayu and Dompu, the positive rate for both *Babesias* was equal, while in Padang Mangateh, the positive rate of *B. bigemina* was higher than that of *B. bovis*. This divergence might be caused by a different distribution of tick vectors in these geographical areas. In Indonesia, *R. microplus*, the common vector of both *B. bovis* and *B. bigemina*, is widespread across the archipelago and has been reported in several islands in Indonesia, such as Sumatera, Java, Kalimantan, Sulawesi, Bali, Lombok and Timor [34]. Other tick species that might transmit *Babesia* parasites, such as *R. annulatus* and *R. geigyi* that exclusively transmit *B. bovis* and *R. decoloratus* and *R. evertsi* that transmit only *B. bigemina*, have not been reported in Indonesia. Therefore, further studies are necessary to determine which tick species are present in the country, possibly providing the reason for the significant difference between *B. bovis* and *B. bigemina* infection rates between different locations.

The ELISA, ICT and nPCR in this study employed the same gene regions. We found a high correlation between ELISA result with *bov*ICT and dual-ICT on *B. bovis* SBP-4 antibody detection, however, there was no correlation between ELISA and nPCR. For *B. bigemina*, the correlations of ELISA result with *big*ICT, dual ICT and nPCR were high. Our results regarding the correlation between methods were similar with the previous study using the same gene regions in Syria, Thailand and Egypt [10, 11, 26], except for nPCR of *B. bovis*. The results also showed that, the positive rates by nPCR for both *Babesia* species were lower than those of ELISA and ICTs. The presence of antibodies for a long period in the blood circulation of host, even after the parasites were already cleared, might explain the discrepancies between those methods [36].

The breeds of cattle also affected the rate of positive samples. Among the breeds of cattle, Pesisir cattle had the highest positive rate of *B. bovis* and *B. bigemina* infections using ELISA and ICTs. This breed also showed the highest positive rate for *B. bigemina* infection, but the lowest for *B. bovis* using nPCR. We also reported the rate of *B. bovis* and *B. bigemina* infection in Bali cattle. Our study is the first report of *Babesia* infection in Pesisir and Bali cattle. It is speculated that Bali cattle are more resistant to *Babesia* infection, but our results showed that positive rates of *B. bovis* were high using serology and molecular detections. Further evaluation of *Babesia* infection in Bali cattle is necessary since the breed is an Indonesian native and had been distributed in many part of the country from their origin location due to their adaptatability to tropical climates and high meat productions. Age groups of cattle also affected the positive rate of *B. bovis* and *B. bigemina*. Young cattle (age < 2 years) had a lower positive rate of *B. bovis* and *B. bigemina* infections compared with older cattle. The difference might be caused by the higher innate immune response of cattle at the young age [11, 35, 37].

The genetic characterization of Indonesian *B. bovis SBP-4* and *B. bigemina RAP-1a* isolates showed a high homology with other isolates in many countries. This result further supports the usefulness of this gene as a specific target for the PCR screening of *B. bovis*. Indonesia is a transcontinental country that is divided into two biogeographical realms, including the Indomalayan and Australasian realms. Each realm is considered to share a common fauna [38]. However, this does not affect the distribution of *B. bovis* in the country. A significant influence was observed in the distribution of *B. bigemina*. Most *B. bigemina RAP-1a* gene sequences from the western part of Indonesia shared high identities with isolates from the Indomalayan realm. The high identities of those sequences were also observed with published *RAP-1a* sequences from other continents, such as Africa and South America. On the other hand, the *B. bigemina RAP-1a* gene sequences from the Nusa Tenggara area (Lombok Timur, Manggarai Timur, Kupang and Malaka), which belongs to the Australasian realm, were more similar to the Bond strain from Australia. Although Tangerang is located in the western part of Indonesia, the *RAP-1a* gene isolate from this location was identical with isolates from the Nusa Tenggara areas because the sample was collected from cattle imported from Australia. Hence, the strategy for controlling the bovine babesiosis in these locations will benefit from the advancement of the Australian approach to tackling the disease. Furthermore, effective control measures should be applied to cattle importation to avoid the introduction of the disease into susceptible herds.

Unlike *B. bovis SBP-4* gene, the high genetic diversity was observed in the nucleotide sequences of Indonesian *B. bovis* ITS region. At least seven different strains of *B. bovis* were distributed in the country. Conversely, the diversity of *B. bigemina* ITS was not as high as *B. bovis*. Only three difference strains were observed in our study. The results were in agreement with other studies using *B. bovis* ITS as a target region in Thailand [27, 39]. Since *B. bovis* has a higher pathogenicity than *B. bigemina*, the correlation of different *B. bovis* strains with their pathogenicity could be explored and characterized in future studies.

## Conclusions

Our study provided the current distribution of bovine babesiosis in Indonesia. The disease is endemic in the country, and effective control strategies are necessary to reduce economic losses. We found that Jombang and Lamongan, while endemicity was very low serologically, currently acquired new infections of bovine babesiosis from other locations. The potency of the outbreaks was high in these locations. We also demonstrated the suitability of our diagnostic methods for use in routine *Babesia* detection in the country. This study presents a basis to explore the epidemiology of bovine babesiosis and the distribution of the tick vector in this country.

## Additional files

Additional file 1: Expected conditional probability of mixed infections between *B. bovis* and *B. bigemina* using ELISA (**Table S1**), *bov*ICT and *big*ICT (**Table S2**), dual-ICT (**Table S3**), and nPCR (**Table S4**). (XLSX 24 kb)

Additional file 2: Alignment of partial amino acid sequences of *Babesia bovis SBP-4* (**A**), partial nucleotide sequences of *B. bovis SBP-4* (**B**), partial amino acid sequences of *B. bigemina RAP-1a* (**C**), partial nucleotide sequences of *B. bigemina RAP-1a* (**D**), nucleotide sequences *B. bovis* ITS region (**E**), and nucleotide sequences *B. bigemina* ITS region (**F**) from Indonesian isolates. (DOCX 27 kb)

Additional file 3: Percentage identities of *Babesia bovis SBP-4* (**Table S5**), *Babesia bigemina RAP-1a* (**Table S6**), *Babesia bovis* ITS region (**Table S7**), and *Babesia bigemina* ITS region (**Table S8**) sequences among Indonesian isolates determined by Clustal Omega alignment [29]. (XLSX 20 kb)

Additional file 4 **Figure S1.** Phylogenetic tree of *Babesia bovis SBP-4* gene sequences. **Figure. S2.** Phylogenetic analysis of *Babesia bigemina RAP-1a* gene sequences. (PDF 170 kb)

## Abbreviations

χ^2^: Chi-square
*big*ICT: ICT for *B. bigemina* detection
BLAST: Basic local alignment search tool
BLASTn: BLAST for nucleotide
*bov*ICT: ICT for *B. bovis* detection
cDNA: Complementary DNA
*df*: Degree of freedom
DNA: Deoxyribonucleic acid
dNTP: Deoxyribose nucleoside triphosphates
dual-ICT: ICT for *B. bovis* and *B. bigemina* detections
ELISA: Enzyme-linked immunosorbent assay
ICT: Immunochromatographic test
iELISA: Indirect ELISA
ITS: Internal transcribed spacer region
nPCR: Nested PCR
PBS: Phosphate buffer saline
PCR: Polymerase chain reaction
RAP-1/CT17: Truncated C-terminal RAP-1
RAP-1a: Rhoptry-associated protein 1a
RBC: Red blood cells
SBP-4: Spherical body protein 4
Se: Sensitivity
Sp: Specificity
