# Supplementary material for: Molecular and serological detection of bovine babesiosis in Indonesia
Source: Parasit Vectors. 2017 Nov 6;10:550. doi: 10.1186/s13071-017-2502-0 (PMC5674684; doi:10.1186/s13071-017-2502-0)
Supplement: Supplementary file 2 — Alignment of partial amino acid sequences of Babesia bovis SBP-4 (A), partial nucleotide sequences of B. bovis SBP-4 (B), partial amino acid sequences of B. bigemina RAP-1a (C), partial nucleotide sequences of B. bigemina RAP-1a (D), nucleotide sequences B. bovis ITS region (E), and nucleotide sequences B. bigemina ITS region (F) from Indonesian isolates. (DOCX 27 kb) [file 13071_2017_2502_MOESM2_ESM.docx]

**Additional file 2: A.** Alignment of partial protein sequences of *B. bovis* sbp4 gene among Indonesian isolates. Amino acid substitutions were indicated by black background. Alignments were inferred by CLUSTAL O (1.2.4) multiple sequence alignment [29].

KY484534.Dompu-146.Indonesia EIPVPEEEQGKEEKTEETETKSEEDGEETEVTEGADEAAPAILHADLQNKFIDEVVVFRN 60

KY562845.Malaka-210.Indonesia EIPVPEEEQGKEEKTEETETKSEEDGEETEGTEGADEAAPAILHADLQNKFIDEVVAFRN 60

KY484530.Mandailing Natal-293.Indonesia EIPVPEEEQGKEEKTEETETKSEEGXX--EGAEGADEAAPAILHADLQNKFIDEVVVFRN 58

KY484523.Tangerang-30.Indonesia EIPVPEEEQGKEEKTEETETKSEEDGE--E-AEGADEAAPAILHADLQNKFIDEVVVFRN 57

KY484524.Bogor-44.Indonesia EIPVPEEEQGKEEKTEETETKSEEDGE--E-AEGADEAAPAILHADLQNKFIDEVVVFRN 57

KY562847.Jombang-479.Indonesia EIPVPEEEQGKEEKTEETETKSEEDGE--E-AEGADEAAPAILHADLQNKFIDEVVVFRN 57

KY484522.Karawang-3.Indonesia EIPVPEEEQGKEEKTEETETKSEEDGE--E-AEGADEAAPAILHADLQNKFIDEVVVFRN 57

KY484525.Indramayu-70.Indonesia EIPVPEEEQGKEEKTEETETKSEEDGE--E-AEGADEAAPAILHADLQNKFIDEVVVFRN 57

KY484527.Lombok Timur-162.Indonesia EIPVPEEEQGKEEKTEETETKSEEDGE--E-AEGADEAAPAILHADLQNKFIDEVVVFRN 57

KY484533.Lamongan-452.Indonesia EIPVPEEEQGKEEKTEETETKSEEDGE--E-AEGADEAAPAILHADLQNKFIDEVVVFRN 57

KY562846.Tabalong-231.Indonesia EIPVPEEEQGKEEKTEETETKSEEDGE--E-AEGADEAAPAILHADLQNKFIDEVVVFRN 57

KY484526.Padang Mangateh-109.Indonesia EIPVPEEEQGKEEKTEETETKSEEDGE--E-AEGADEAAPAILHADLQNKFIDEVVVFRN 57

KY484528.Kupang-170.Indonesia EIPVPEEEQGKEEKTEETETKSEEDGE--E-AEGADEAAPAILHADLQNKFIDEVVVFRN 57

KY484529.Manggarai Timur-188.Indonesia EIPVPEEEQGKEEKTEETETKSEEDGE--E-AEGADEAAPAILHADLQNKFIDEVVVFRN 57

KY484531.Tapanuli Selatan-337.Indonesia EIPVPEEEQGKEEKTEETETKSEEDGE--E-AEGADEAAPAILHADLQNKFIDEVVVFRN 57

KY484532.Bulukumba-364.Indonesia EIPVPEEEQGKEEKTEETETKSEEDGE--E-AEGADEAAPAILHADLQNKFIDEVVVFRN 57

************************. * :************************.***

KY484534.Dompu-146.Indonesia CFDTAVSVNVDGKQIYFTATGNEAEDFVEVEADKYEKFMNKIAKTFTIDVSETDLKSECL 120

KY562845.Malaka-210.Indonesia CFDTAVSVNVDGKQIYFTATGNEAEDFVEVEADKYEKFMNKIAKTFTIDVSETDLKSECL 120

KY484530.Mandailing Natal-293.Indonesia CFDTAVSVNVDGKQIYFTATGNEAENFEEVEADEYEKFMNKIAKTFTIDISETDLKSECL 118

KY484523.Tangerang-30.Indonesia CFDTAVSVNVDGKQIYFTATGNEAEDFEEVEADEYEKFMNKIAKTFTIDISETDLKSECL 117

KY484524.Bogor-44.Indonesia CFDTAVSVNVDGKQIYFTATGNEAEDFEEVEADEYEKFMNKIAKTFTIDISETDLKSECL 117

KY562847.Jombang-479.Indonesia CFDTAVSVNVDGKQIYFTATGNEAEDFEEVEADEYEKFMNKIAKTFTIDISETDLKSECL 117

KY484522.Karawang-3.Indonesia CFDTAVSVNVDGKQIYFTATGNEAEDFEEVEADEYEKFMNKIAKTFTIDISETDLKSECL 117

KY484525.Indramayu-70.Indonesia CFDTAVSVNVDGKQIYFTATGNEAEDFEEVEADEYEKFMNKIAKTFTIDISETDLKSECL 117

KY484527.Lombok Timur-162.Indonesia CFDTAVSVNVDGKQIYFTATGNEAEDFEEVEADEYEKFMNKIAKTFTIDISETDLKSECL 117

KY484533.Lamongan-452.Indonesia CFDTAVSVNVDGKQIYFTATGNEAEDFEEVEADEYEKFMNKIAKTFTIDISETDLKSECL 117

KY562846.Tabalong-231.Indonesia CFDTAVSVNVDGKQIYFTATGNEAEDFEEVEADEYKKFMNKIAKTFTIDISETDLKSECL 117

KY484526.Padang Mangateh-109.Indonesia CFDTAVSVNVDGKQIYFTATGNEAEDFEEVEADEYEKFMNKIAKTFTIDISETDLKSECL 117

KY484528.Kupang-170.Indonesia CFDTAVSVNVDGKQIYFTATGNEAEDFEEVEADEYEKFMNKIAKTFTIDISETDLKSECL 117

KY484529.Manggarai Timur-188.Indonesia CFDTAVSVNVDGKQIYFTATGNEAEDFEEVEADEYEKFMNKIAKTFTIDISETDLKSECL 117

KY484531.Tapanuli Selatan-337.Indonesia CFDTAVSVNVDGKQIYFTATGNEAEDFEEVEADEYEKFMNKIAKTFTIDISETDLKSECL 117

KY484532.Bulukumba-364.Indonesia CFDTAVSVNVDGKQIYFTATGNEAEDFEEVEADEYEKFMNKIAKTFTIDISETDLKSECL 117

*************************:* *****:*:*************:**********

KY484534.Dompu-146.Indonesia VAEEDKVYDLSWYSIHAAPCYLADKVLFKGNPLWEAADKKEHFGGCSVIN 170

KY562845.Malaka-210.Indonesia VAEEDKVYDLSWYSIHAAPCYLADKVLFKGNPLWEAADKKEHFGGCSVIN 170

KY484530.Mandailing Natal-293.Indonesia VSEDDKVYDLSWYSIHAAPCYLADKVLFKGNPLWEAADKKEHFGGCSVIN 168

KY484523.Tangerang-30.Indonesia VAEDDKVYDLSWYSIHAAPCYLADKVLFKGNPLWEATDKKEHFAGCSVIN 167

KY484524.Bogor-44.Indonesia VAEDDKVYDLSWYSIHAAPCYLADIVLFKGNALWEATDKKEHFAGCSVIN 167

KY562847.Jombang-479.Indonesia VAADDKVYDPSWYSIHAAPCYLADKVLFKGNPLWEATDKKEHFAGCSVIN 167

KY484522.Karawang-3.Indonesia VAEDDKVYDLSWYSIHAAPCYLADKVLFKGNPLWEATDKKEHSAGCSVIN 167

KY484525.Indramayu-70.Indonesia VAEDDKVYDLSWYSIHAAPCYLADKVLFKGNPLWEATDKKEHSAGCSVIN 167

KY484527.Lombok Timur-162.Indonesia VAEDDKVYDLSWYSIHAAPCYLADKVLFKGNPLWEATDKEEHFAGCSVIN 167

KY484533.Lamongan-452.Indonesia VAEDDKVYDLSRYSIHAAPCYLADKVLFKGNPLWEATDKKEHFAGCSVIN 167

KY562846.Tabalong-231.Indonesia VAEDDKVYDLSWYSIHAAPCYLADKVLFKGNPLWEATDKKEHFAGCSVIN 167

KY484526.Padang Mangateh-109.Indonesia VAEDDKVYDLSWYSIHAAPCYLADKVLFKGNPLWEATDKKEHFAGCSVIN 167

KY484528.Kupang-170.Indonesia VAEDDKVYDLSWYSIHAAPCYLADKVLFKGNPLWEATDKKEHFAGCSVIN 167

KY484529.Manggarai Timur-188.Indonesia VAEDDKVYDLSWYSIHAAPCYLADKVLFKGNPLWEATDKKEHFAGCSVIN 167

KY484531.Tapanuli Selatan-337.Indonesia VAEDDKVYDLSWYSIHAAPCYLADKVLFKGNPLWEATDKKEHFAGCSVIN 167

KY484532.Bulukumba-364.Indonesia VAEDDKVYDLSWYSIHAAPCYLADKVLFKGNPLWEATDKKEHFAGCSVIN 167

*: :***** * ************ ****** ****:**:** .******

**Additional file 2: B.** Alignment of partial nucleotide sequences of *B. bovis* sbp4 gene among Indonesian isolates. Nucleotide substitutions were indicated green background. The alignment was inferred by CLUSTAL O(1.2.4) multiple sequence alignment.

KY484534.Dompu-146.Indonesia GAAATCCCTGTTCCAGAGGAAGAGCAAGGCAAAGAGGAGAAGACTGAGGAGACTGAGACC 60

KY562845.Malaka-210.Indonesia GAAATCCCTGTTCCAGAGGAGGAGCAAGGCAAAGAGGAGAAGACCGAGGAGACTGAGACC 60

KY484530.Mandailing Natal-293.Indonesia GAAATCCCTGTTCCAGAGGAAGAGCAAGGCAAAGAGGAGAAGACTGAGGAGACTGAGACC 60

KY484524.Bogor-44.Indonesia GAAATCCCTGTTCCGGAGGAAGAGCAAGGCAAAGAGGAGAAGACTGAGGAGACTGAGACC 60

KY562847.Jombang-479.Indonesia GAAATCCCTGTTCCAGAGGAAGAGCAAGGCAAAGAGGAGAAGACTGAGGAGACTGAGACC 60

KY484523.Tangerang-30.Indonesia GAAATCCCTGTTCCAGAGCAAGAGCAAGGCAAAGAGGAGAAGACTGAGGAGACTGAGACC 60

KY484528.Kupang-170.Indonesia GAAATCCCTGTTCCAGAGGAAGAGCAAGGCAAAGAGGAGAAGACTGAGGAGACTGAGACC 60

KY484533.Lamongan-452.Indonesia GAAATCCCTGTTCCAGAGGAAGAGCAAGGCAAAGAGGAGAAGACTGAGGAGACTGAGACC 60

KY484522.Karawang-3.Indonesia GAAATCCCTGTTCCAGAGGAAGAGCAAGGCAAAGAGGAGAAGACTGAGGAGACTGAGACC 60

KY484525.Indramayu-70.Indonesia GAAATCCCTGTTCCAGAGGAAGAGCAAGGCAAAGAGGAGAAGACTGAGGAGACTGAGACC 60

KY484527.Lombok Timur-162.Indonesia GAAATCCCTGTTCCAGAGGAAGAGCAAGGCAAAGAGGAGAAGACTGAGGAGACTGAGACC 60

KY562846.Tabalong-231.Indonesia GAAATCCCTGTTCCAGAGGAAGAGCAAGGCAAAGAGGAGAAGACTGAGGAGACTGAGACC 60

KY484526.Padang Mangateh-109.Indonesia GAAATCCCTGTTCCAGAGGAAGAGCAAGGCAAAGAGGAGAAGACTGAGGAGACTGAGACC 60

KY484529.Manggarai Timur-188.Indonesia GAAATCCCTGTTCCAGAGGAAGAGCAAGGCAAAGAGGAGAAGACTGAGGAGACTGAGACC 60

KY484531.Tapanuli Selatan-337.Indonesia GAAATCCCTGTTCCAGAGGAAGAGCAAGGCAAAGAGGAGAAGACTGAGGAGACTGAGACC 60

KY484532.Bulukumba-364.Indonesia GAAATCCCTGTTCCAGAGGAAGAGCAAGGCAAAGAGGAGAAGACTGAGGAGACTGAGACC 60

****************** * *********************** ***************

KY484534.Dompu-146.Indonesia AAGTCAGAAGAGGATGGAGAGGAAACCGAGGTAACCGAGGGCGCTGATGAAGCCGCCCCC 120

KY562845.Malaka-210.Indonesia AAGTCAGAAGAGGATGGAGAGGAAACCGAGGGAACCGAGGGCGCTGATGAAGCCGCCCCC 120

KY484530.Mandailing Natal-293.Indonesia AAGTCAG---------AAGAGGGTGCCGAGGGCGCCGAGGGCGCTGATGAAGCCGCCCCC 111

KY484524.Bogor-44.Indonesia AAGTCAG---------AAGAGGATGGAGAGGAAGCCGAGGGCGCTGATGAAGCCGCCCCC 111

KY562847.Jombang-479.Indonesia AAGTCAG---------AAGAGGATGGGGAGGAAGCCGAGGGCGCTGATGAAGCCGCCCCC 111

KY484523.Tangerang-30.Indonesia AAGTCAG---------AAGAGGATGGAGAGGAAGCCGAGGGCGCTGATGAAGCCGCCCCC 111

KY484528.Kupang-170.Indonesia AAGTCAG---------AAGAGGATGGAGAGGAAGCCGAGGGCGCTGATGAAGCCGCCCCC 111

KY484533.Lamongan-452.Indonesia AAGTCAG---------AAGAGGATGGGGAGGAAGCCGAGGGCGCTGATGAAGCCGCCCCC 111

KY484522.Karawang-3.Indonesia AAGTCAG---------AAGAGGATGGAGAGGAAGCCGAGGGCGCTGATGAAGCCGCCCCC 111

KY484525.Indramayu-70.Indonesia AAGTCAG---------AAGAGGATGGAGAGGAAGCCGAGGGCGCTGATGAAGCCGCCCCC 111

KY484527.Lombok Timur-162.Indonesia AAGTCAG---------AAGAGGATGGAGAGGAAGCCGAGGGCGCTGATGAAGCCGCCCCC 111

KY562846.Tabalong-231.Indonesia AAGTCAG---------AAGAGGATGGAGAGGAAGCCGAGGGCGCTGATGAAGCCGCCCCC 111

KY484526.Padang Mangateh-109.Indonesia AAGTCAG---------AAGAGGATGGAGAGGAAGCCGAGGGCGCTGATGAAGCCGCCCCC 111

KY484529.Manggarai Timur-188.Indonesia AAGTCAG---------AAGAGGATGGAGAGGAAGCCGAGGGCGCTGATGAAGCCGCCCCC 111

KY484531.Tapanuli Selatan-337.Indonesia AAGTCAG---------AAGAGGATGGAGAGGAAGCCGAGGGCGCTGATGAAGCCGCCCCC 111

KY484532.Bulukumba-364.Indonesia AAGTCAG---------AAGAGGATGGAGAGGAAGCCGAGGGCGCTGATGAAGCCGCCCCC 111

******* ***** **** **************************

KY484534.Dompu-146.Indonesia GCTATACTCCACGCTGATCTCCAGAACAAGTTCATTGATGAAGTAGTTGTTTTCCGCAAC 180

KY562845.Malaka-210.Indonesia GCTATACTCCACGCTGATCTCCAGAACAAGTTCATTGATGAAGTAGTTGCTTTCCGCAAC 180

KY484530.Mandailing Natal-293.Indonesia GCTATACTTCACGCTGATCTCCAGAACAAGTTCATTGATGAAGTAGTTGTTTTCCGCAAC 171

KY484524.Bogor-44.Indonesia GCTATACTCCACGCTGACCTCCAGAACAAGTTCATTGATGAAGTAGTTGTTTTCCGCAAT 171

KY562847.Jombang-479.Indonesia GCTATACTCCACGCTGACCTCCAGAACAAGTTCATTGATGAAGTAGTTGTTTTCCGCAAT 171

KY484523.Tangerang-30.Indonesia GCTATACTCCACGCTGACCTCCAGAACAAGTTCATTGATGAAGTAGTTGTTTTCCGCAAT 171

KY484528.Kupang-170.Indonesia GCTATACTCCACGCTGACCTCCAGAACAAGTTCATTGATGAAGTAGTTGTTTTCCGCAAT 171

KY484533.Lamongan-452.Indonesia GCTATACTCCACGCTGACCTCCAGAACAAGTTCATTGATGAAGTAGTTGTTTTCCGCAAT 171

KY484522.Karawang-3.Indonesia GCTATACTCCACGCTGACCTCCAGAACAAGTTCATTGATGAAGTAGTTGTTTTCCGCAAT 171

KY484525.Indramayu-70.Indonesia GCTATACTCCACGCTGACCTCCAGAACAAGTTCATTGATGAAGTAGTTGTTTTCCGCAAT 171

KY484527.Lombok Timur-162.Indonesia GCTATACTCCACGCTGACCTCCAGAACAAGTTCATTGATGAAGTAGTTGTTTTCCGCAAT 171

KY562846.Tabalong-231.Indonesia GCTATACTCCACGCTGACCTCCAGAACAAGTTCATTGATGAAGTAGTTGTTTTCCGCAAT 171

KY484526.Padang Mangateh-109.Indonesia GCTATACTCCACGCTGACCTCCAGAACAAGTTCATTGATGAAGTAGTTGTTTTCCGCAAT 171

KY484529.Manggarai Timur-188.Indonesia GCTATACTCCACGCTGACCTCCAGAACAAGTTCATTGATGAAGTAGTTGTTTTCCGCAAT 171

KY484531.Tapanuli Selatan-337.Indonesia GCTATACTCCACGCTGACCTCCAGAACAAGTTCATTGATGAAGTAGTTGTTTTCCGCAAT 171

KY484532.Bulukumba-364.Indonesia GCTATACTCCACGCTGACCTCCAGAACAAGTTCATTGATGAAGTAGTTGTTTTCCGCAAT 171

******** ******** ******************************* *********

KY484534.Dompu-146.Indonesia TGCTTTGACACCGCTGTTTCTGTCAATGTCGATGGTAAGCAGATCTACTTCACTGCCACT 240

KY562845.Malaka-210.Indonesia TGCTTTGACACCGCTGTTTCTGTCAATGTCGATGGTAAGCAGATCTACTTCACTGCCACT 240

KY484530.Mandailing Natal-293.Indonesia TGCTTTGACACCGCTGTTTCCGTCAATGTCGATGGTAAGCAGATCTACTTCACTGCCACT 231

KY484524.Bogor-44.Indonesia TGCTTTGACACCGCTGTTTCCGTCAATGTCGATGGTAAGCAGATCTACTTCACTGCCACT 231

KY562847.Jombang-479.Indonesia TGCTTTGACACCGCTGTTTCCGTCAATGTCGATGGTAAGCAGATCTACTTCACTGCCACT 231

KY484523.Tangerang-30.Indonesia TGCTTTGACACCGCTGTTTCCGTCAATGTCGATGGTAAGCAGATCTACTTCACTGCCACT 231

KY484528.Kupang-170.Indonesia TGCTTCGACACCGCTGTTTCCGTCAATGTCGATGGTAAGCAGATCTACTTCACTGCCACT 231

KY484533.Lamongan-452.Indonesia TGCTTTGACACCGCTGTTTCCGTCAATGTCGATGGTAAGCAGATCTACTTCACTGCCACT 231

KY484522.Karawang-3.Indonesia TGCTTTGACACCGCTGTTTCCGTCAATGTCGATGGTAAGCAGATCTACTTCACTGCCACT 231

KY484525.Indramayu-70.Indonesia TGCTTTGACACCGCTGTTTCCGTCAATGTCGATGGTAAGCAGATCTACTTCACTGCCACT 231

KY484527.Lombok Timur-162.Indonesia TGCTTTGACACCGCTGTTTCCGTCAATGTCGATGGTAAGCAGATCTACTTCACTGCCACT 231

KY562846.Tabalong-231.Indonesia TGCTTTGACACCGCTGTTTCCGTCAATGTCGATGGTAAGCAGATCTACTTCACTGCCACT 231

KY484526.Padang Mangateh-109.Indonesia TGCTTTGACACCGCTGTTTCCGTCAATGTCGATGGTAAGCAGATCTACTTCACTGCCACT 231

KY484529.Manggarai Timur-188.Indonesia TGCTTTGACACCGCTGTTTCCGTCAATGTCGATGGTAAGCAGATCTACTTCACTGCCACT 231

KY484531.Tapanuli Selatan-337.Indonesia TGCTTTGACACCGCTGTTTCCGTCAATGTCGATGGTAAGCAGATCTACTTCACTGCCACT 231

KY484532.Bulukumba-364.Indonesia TGCTTTGACACCGCTGTTTCCGTCAATGTCGATGGTAAGCAGATCTACTTCACTGCCACT 231

***** ************** ***************************************

KY484534.Dompu-146.Indonesia GGCAATGAAGCCGAGGACTTCGTGGAGGTTGAGGCAGATAAGTACGAGAAGTTCATGAAC 300

KY562845.Malaka-210.Indonesia GGCAATGAAGCCGAGGACTTCGTGGAGGTTGAGGCAGATAAGTACGAGAAGTTCATGAAC 300

KY484530.Mandailing Natal-293.Indonesia GGCAATGAAGCCGAGAACTTCGAGGAGGTTGAGGCCGATGAATACGAGAAGTTCATGAAC 291

KY484524.Bogor-44.Indonesia GGCAATGAAGCCGAGGACTTCGAGGAGGTTGAGGCCGATGAATACGAGAAGTTCATGAAC 291

KY562847.Jombang-479.Indonesia GGCAATGAAGCCGAGGACTTCGAGGAGGTTGAGGCCGATGAATACGAGAAGTTCATGAAC 291

KY484523.Tangerang-30.Indonesia GGCAATGAAGCCGAGGACTTCGAGGAGGTTGAGGCCGATGAATACGAGAAGTTCATGAAC 291

KY484528.Kupang-170.Indonesia GGCAATGAAGCCGAGGACTTCGAGGAGGTTGAGGCCGATGAATACGAGAAGTTCATGAAC 291

KY484533.Lamongan-452.Indonesia GGCAATGAAGCCGAGGACTTCGAGGAGGTTGAGGCCGATGAATACGAGAAGTTCATGAAC 291

KY484522.Karawang-3.Indonesia GGCAATGAAGCCGAGGACTTCGAGGAGGTTGAGGCCGATGAATACGAGAAGTTCATGAAC 291

KY484525.Indramayu-70.Indonesia GGCAATGAAGCCGAGGACTTCGAGGAGGTTGAGGCCGATGAATACGAGAAGTTCATGAAC 291

KY484527.Lombok Timur-162.Indonesia GGCAATGAAGCCGAGGACTTCGAGGAGGTTGAGGCCGATGAATACGAGAAGTTCATGAAC 291

KY562846.Tabalong-231.Indonesia GGCAATGAAGCCGAGGACTTCGAGGAGGTTGAGGCCGATGAATACAAGAAGTTCATGAAC 291

KY484526.Padang Mangateh-109.Indonesia GGCAATGAAGCCGAGGACTTCGAGGAGGTTGAGGCCGATGAATACGAGAAGTTCATGAAC 291

KY484529.Manggarai Timur-188.Indonesia GGCAATGAAGCCGAGGACTTCGAGGAGGTTGAGGCCGATGAATACGAGAAGTTCATGAAC 291

KY484531.Tapanuli Selatan-337.Indonesia GGCAATGAAGCCGAGGACTTCGAGGAGGTTGAGGCCGATGAATACGAGAAGTTCATGAAC 291

KY484532.Bulukumba-364.Indonesia GGCAATGAAGCCGAGGACTTCGAGGAGGTTGAGGCCGATGAATACGAGAAGTTCATGAAC 291

*************** ****** ************ *** * *** **************

KY484534.Dompu-146.Indonesia AAGATTGCCAAGACTTTCACTATTGACGTTAGTGAAACCGATCTTAAGAGCGAATGCCTT 360

KY562845.Malaka-210.Indonesia AAGATTGCCAAGACTTTCACTATTGACGTTAGTGAAACCGATCTTAAGAGCGAATGCCTT 360

KY484530.Mandailing Natal-293.Indonesia AAGATTGCCAAGACCTTCACTATTGACATCAGTGAAACCGATCTCAAGAGCGAATGCCTT 351

KY484524.Bogor-44.Indonesia AAGATTGCCAAGACCTTCACTATTGACATCAGTGAAACCGATCTTAAGAGCGAATGCCTT 351

KY562847.Jombang-479.Indonesia AAGATTGCCAAGACCTTCACTATTGACATCAGTGAAACCGATCTTAAGAGCGAATGCCTT 351

KY484523.Tangerang-30.Indonesia AAGATTGCCAAGACCTTCACTATTGACATCAGTGAAACCGATCTTAAGAGCGAATGCCTT 351

KY484528.Kupang-170.Indonesia AAGATTGCCAAGACCTTCACTATTGACATCAGTGAAACCGATCTTAAGAGCGAATGCCTT 351

KY484533.Lamongan-452.Indonesia AAGATTGCCAAGACCTTCACTATTGACATCAGTGAAACCGATCTTAAGAGCGAATGCCTT 351

KY484522.Karawang-3.Indonesia AAGATTGCCAAGACCTTCACTATTGACATCAGTGAAACCGATCTTAAGAGCGAATGCCTT 351

KY484525.Indramayu-70.Indonesia AAGATTGCCAAGACCTTCACTATTGACATCAGTGAAACCGATCTTAAGAGCGAATGCCTT 351

KY484527.Lombok Timur-162.Indonesia AAGATTGCCAAGACCTTCACTATTGACATCAGTGAAACCGATCTTAAGAGCGAATGCCTT 351

KY562846.Tabalong-231.Indonesia AAGATTGCCAAGACCTTCACTATTGACATCAGTGAAACCGATCTTAAGAGCGAATGCCTT 351

KY484526.Padang Mangateh-109.Indonesia AAGATTGCCAAGACCTTCACTATTGACATCAGTGAAACCGATCTTAAGAGCGAATGCCTT 351

KY484529.Manggarai Timur-188.Indonesia AAGATTGCCAAGACCTTCACTATTGACATCAGTGAAACCGATCTTAAGAGCGAATGCCTT 351

KY484531.Tapanuli Selatan-337.Indonesia AAGATTGCCAAGACCTTCACTATTGACATCAGTGAAACCGATCTTAAGAGCGAATGCCTT 351

KY484532.Bulukumba-364.Indonesia AAGATTGCCAAGACCTTCACTATTGACATCAGTGAAACCGATCTTAAGAGCGAATGCCTT 351

************** ************ * ************** ***************

KY484534.Dompu-146.Indonesia GTTGCCGAGGAAGACAAGGTATACGACTTGTCATGGTACAGCATCCACGCTGCCCCCTGC 420

KY562845.Malaka-210.Indonesia GTTGCCGAGGAAGACAAGGTATACGACTTGTCATGGTACAGCATCCACGCTGCCCCCTGC 420

KY484530.Mandailing Natal-293.Indonesia GTTTCTGAGGACGACAAGGTATACGACTTGTCATGGTACAGCATCCACGCTGCCCCCTGC 411

KY484524.Bogor-44.Indonesia GTTGCTGAGGACGACAAGGTATACGACCTGTCATGGTACAGCATCCACGCTGCCCCCTGC 411

KY562847.Jombang-479.Indonesia GTTGCTGCGGACGACAAGGTATACGACCCGTCATGGTACAGCATCCACGCTGCCCCCTGC 411

KY484523.Tangerang-30.Indonesia GTTGCTGAGGACGACAAGGTATACGACCTGTCATGGTACAGCATCCACGCTGCCCCCTGC 411

KY484528.Kupang-170.Indonesia GTTGCTGAGGACGACAAGGTATACGACCTGTCCTGGTACAGCATCCACGCTGCCCCCTGC 411

KY484533.Lamongan-452.Indonesia GTTGCTGAGGACGACAAGGTATACGACCTGTCACGGTACAGCATCCACGCTGCCCCCTGC 411

KY484522.Karawang-3.Indonesia GTTGCTGAGGACGACAAGGTATACGACCTGTCATGGTACAGCATCCACGCTGCCCCCTGC 411

KY484525.Indramayu-70.Indonesia GTTGCTGAGGACGACAAGGTATACGACCTGTCATGGTACAGCATCCACGCTGCCCCCTGC 411

KY484527.Lombok Timur-162.Indonesia GTTGCTGAGGACGACAAGGTATACGACCTGTCATGGTACAGCATCCACGCTGCCCCCTGC 411

KY562846.Tabalong-231.Indonesia GTTGCTGAGGACGACAAGGTATACGACCTGTCATGGTACAGCATCCACGCTGCCCCCTGC 411

KY484526.Padang Mangateh-109.Indonesia GTTGCTGAGGACGACAAGGTATACGACCTGTCATGGTACAGCATCCACGCTGCCCCCTGC 411

KY484529.Manggarai Timur-188.Indonesia GTTGCTGAGGACGACAAGGTATACGACCTGTCATGGTACAGCATCCACGCTGCCCCCTGC 411

KY484531.Tapanuli Selatan-337.Indonesia GTTGCTGAGGACGACAAGGTATACGACCTGTCATGGTACAGCATCCACGCTGCCCCCTGC 411

KY484532.Bulukumba-364.Indonesia GTTGCTGAGGACGACAAGGTATACGACCTGTCATGGTACAGCATCCACGCTGCCCCCTGC 411

*** * * *** *************** *** **************************

KY484534.Dompu-146.Indonesia TACCTCGCTGACAAGGTCTTGTTCAAGGGCAACCCTCTCTGGGAGGCTGCTGACAAGAAG 480

KY562845.Malaka-210.Indonesia TACCTCGCTGACAAGGTCTTGTTCAAGGGCAACCCTCTCTGGGAGGCTGCTGACAAGAAG 480

KY484530.Mandailing Natal-293.Indonesia TACCTCGCTGACAAGGTCTTGTTCAAGGGCAACCCTCTCTGGGAGGCTGCTGACAAGAAG 471

KY484524.Bogor-44.Indonesia TACCTCGCCGACATCGTCTTGTTCAAGGGCAACGCTCTCTGGGAGGCCACTGACAAGAAG 471

KY562847.Jombang-479.Indonesia TACCTCGCCGACAAGGTCTTGTTCAAGGGCAACCCTCTCTGGGAGGCCACTGACAAGAAG 471

KY484523.Tangerang-30.Indonesia TACCTCGCCGACAAGGTCTTGTTCAAGGGCAACCCTCTCTGGGAGGCCACTGACAAGAAG 471

KY484528.Kupang-170.Indonesia TACCTCGCCGACAAGGTCTTGTTCAAGGGCAACCCTCTCTGGGAGGCCACTGACAAGAAG 471

KY484533.Lamongan-452.Indonesia TACCTCGCCGACAAGGTCTTGTTCAAGGGCAACCCTCTCTGGGAGGCCACTGACAAGAAG 471

KY484522.Karawang-3.Indonesia TACCTCGCCGACAAGGTCTTGTTCAAGGGCAACCCTCTCTGGGAGGCCACTGACAAGAAG 471

KY484525.Indramayu-70.Indonesia TACCTCGCCGACAAGGTCTTGTTCAAGGGCAACCCTCTCTGGGAGGCCACTGACAAGAAG 471

KY484527.Lombok Timur-162.Indonesia TACCTCGCCGACAAGGTCTTGTTCAAGGGCAACCCTCTCTGGGAGGCCACTGACAAGGAG 471

KY562846.Tabalong-231.Indonesia TACCTCGCCGACAAGGTCTTGTTCAAGGGCAACCCTCTCTGGGAGGCCACTGACAAGAAG 471

KY484526.Padang Mangateh-109.Indonesia TACCTCGCCGACAAGGTCTTGTTCAAGGGCAACCCTCTCTGGGAGGCCACTGACAAGAAG 471

KY484529.Manggarai Timur-188.Indonesia TACCTCGCCGACAAGGTCTTGTTCAAGGGCAACCCTCTCTGGGAGGCCACTGACAAGAAG 471

KY484531.Tapanuli Selatan-337.Indonesia TACCTCGCCGACAAGGTCTTGTTCAAGGGCAACCCTCTCTGGGAGGCCACTGACAAGAAG 471

KY484532.Bulukumba-364.Indonesia TACCTCGCCGACAAGGTCTTGTTCAAGGGCAACCCTCTCTGGGAGGCCACTGACAAGAAG 471

******** **** ****************** ************* ******** **

KY484534.Dompu-146.Indonesia GAACACTTTGGAGGTTGCAGTGTTATCAACGA 512

KY562845.Malaka-210.Indonesia GAACACTTTGGAGGTTGCAGTGTTATCAACGA 512

KY484530.Mandailing Natal-293.Indonesia GAACACTTTGGAGGTTGCAGTGTTATCAACGA 503

KY484524.Bogor-44.Indonesia GAGCACTTCGCTGGTTGCAGTGTTATCAACGA 503

KY562847.Jombang-479.Indonesia GAGCACTTCGCTGGTTGCAGTGTTATCAACGA 503

KY484523.Tangerang-30.Indonesia GAGCACTTCGCTGGTTGCAGTGTTATCAACGA 503

KY484528.Kupang-170.Indonesia GAGCACTTCGCTGGTTGCAGTGTTATCAACGA 503

KY484533.Lamongan-452.Indonesia GAGCACTTCGCTGGTTGCAGTGTTATCAACGA 503

KY484522.Karawang-3.Indonesia GAGCACTCCGCTGGTTGCAGTGTTATCAACGA 503

KY484525.Indramayu-70.Indonesia GAGCACTCCGCTGGTTGCAGTGTTATCAACGA 503

KY484527.Lombok Timur-162.Indonesia GAGCACTTCGCTGGTTGCAGTGTTATCAACGA 503

KY562846.Tabalong-231.Indonesia GAGCACTTCGCTGGTTGCAGTGTTATCAACGA 503

KY484526.Padang Mangateh-109.Indonesia GAGCACTTCGCTGGTTGCAGTGTTATCAACGA 503

KY484529.Manggarai Timur-188.Indonesia GAGCACTTCGCTGGTTGCAGTGTTATCAACGA 503

KY484531.Tapanuli Selatan-337.Indonesia GAGCACTTCGCTGGTTGCAGTGTTATCAACGA 503

KY484532.Bulukumba-364.Indonesia GAGCACTTCGCTGGTTGCAGTGTTATCAACGA 503

** **** * ********************

**Additional file 2: C.** Alignment of partial protein sequences of *B. bigemina* rap-1a gene among Indonesian isolates. Amino acid substitutions were indicated by black background. The alignment was inferred by CLUSTAL O(1.2.4) multiple sequence alignment.

KY484512.Padang Mangateh-84.Indonesia SLLSQLACSVSVSRRR*SVWLGATFPLTLEPTLRPPSAK*LAATAST**PRCLR*PRSLS 55

KY484519.Bulukumba-366.Indonesia SLLSQLACSVSVSRRR*SVWLGATFPLTLEPTLRPPSAK*LAATAST**PRCLR*PRSLS 55

KY484521.Jombang-480.Indonesia SLLSQLACSVSVSRRR*SVWLGATFPLTLEPTLRPPSAK*LAATAST**PRCLR*PRSLS 55

KY484510.Karawang-6.Indonesia SLLSQLACSVSVSRRR*SVWLGATFPLTLEPTLRPPSAK*LAATAST**PRCLR*PRSLS 55

KY484520.Lamongan-448.Indonesia SLLSQLACSVSVSRRR*SVWLGATFPLTLEPTLRPPSAK*LAATAST**PRCLR*PRSLS 55

KY484518.Mandailing Natal-290.Indonesia SLLSQLACSVSVSRRR*SVWLGATFPLTLEPTLRPPSAK*LAATAST**PRCLR*PRSLS 55

KY562848.Bogor-43.Indonesia SLLSQLACSVSVSRRR*SVWLGATFPLTLEPTLRPPSAK*LAATAST**PRCLR*PRSLS 55

KY484511.Tangerang-38.Indonesia SLLSQLACSVSVSRGR*SVWLGATFPLTLEPTLRPPSAR*LAATANT**PRCLR*PRSLS 55

KY484513.Lombok Timur-160.Indonesia SLLSQLACSVSVSRGR*SVWLGATFPLTLEPTLRPPSAR*LAATANT**PRCLR*PRSLS 55

KY484515.Manggarai Timur-192.Indonesia SLLSQLACSVSVSRGR*SVWLGATFPLTLEPTLRPPSAR*LAATANT**PRCLR*PRSLS 55

KY484517.Tabalong-239.Indonesia SLLSQLACSVSVSRGR*SVWLGATFPLTLEPTLRPPSAR*LAATANT**PRCLR*PRSLS 55

KY484514.Kupang-181.Indonesia SLLSQLACSVSVSRGR*SVWLGATFPLTLEPTLRPPSGR*IAVTANT**PRCLR*PRSLS 55

KY484516.Malaka-209.Indonesia SLLSQLACSISVSRGR*SVWLGATFPLTLEPTLRPPSGR*IAVTANT**PRCLR*PRSLS 55

*********:**** **********************.:*:*.**.**************

KY484512.Padang Mangateh-84.Indonesia VSPRWLLRLCWLPSATTSICPRTRGGTGSSRSSL*TSLLTLPS*L*STSLSL*RLPTQSW 111

KY484519.Bulukumba-366.Indonesia VSPRWLLRLCWLPSATTSICPRTRGGTGSSRSSL*TSLLTLPS*L*STSLSL*RLPTQSW 111

KY484521.Jombang-480.Indonesia VSPRWLLRLCWLPSATTSICPRTRGGTGSSRSSL*ASLLTLPS*L*STSLSL*RLPTQSW 111

KY484510.Karawang-6.Indonesia VSPRWLLRLCWLPSATTSICPRTRGGTGSSRSSL*TSLLTLPS*L*STSLSL*RLPTQSW 111

KY484520.Lamongan-448.Indonesia VSPRWLLRLCWLPSATTSICPRTRGGTGSSRSSL*TSLLTLPS*L*STSLSL*RLPTQSW 111

KY484518.Mandailing Natal-290.Indonesia VSPRWLLRLCWLPSATTSICPRTRGGTGSSRSSL*TSLLTLPS*L*STSLSL*RLPTQSW 111

KY562848.Bogor-43.Indonesia VSPRWLLRLCWLPSATTSICPRTRGGTGSSRSSL*TSLLTLPS*L*STSLSL*RLPTQSW 111

KY484511.Tangerang-38.Indonesia VSPRWLLRLCWLPSATTSGCPRTRGGTGSSRSSL*PSLPTLPS*F*STSLSL*RLRTQSW 111

KY484513.Lombok Timur-160.Indonesia VSPRWLLRLCWLPSATTSGCPRTRGGTGSSRSSL*PSLPTLPS*F*STSLSL*RLRTQSW 111

KY484515.Manggarai Timur-192.Indonesia VSPRWLLRLCWLPSATTSGCPRTRGGTGSSRSSL*PSLPTLPS*F*STSLSL*RLRTQSW 111

KY484517.Tabalong-239.Indonesia VSPRWLLRLCWLPSAATSGCPRTRGGTGSSRSSL*PSLPTLPS*FRSTSLSL*GLRTQSW 112

KY484514.Kupang-181.Indonesia VYPRWVLRLCWLPSATTSGCPRTRGGTGSSRSSL*PSLPTLPS*F*STSLSLGRLRTQSW 112

KY484516.Malaka-209.Indonesia VSPRWLLRLCWLPSATTSGCPRTRGGTGSSRSSL*PSLPTLPS*F*STSLSLGRLRTQSW 112

* ***:*********:** **************** ** *****: ****** * ****

KY484512.Padang Mangateh-84.Indonesia SLKSTGMLSGMSSVKAP 128

KY484519.Bulukumba-366.Indonesia SLKSAGRLSGMSSVKAP 128

KY484521.Jombang-480.Indonesia SLKSTGRLSGMSSVKAP 128

KY484510.Karawang-6.Indonesia SLKSTGRLSGMSSVKAP 128

KY484520.Lamongan-448.Indonesia SLKSTGRLSGMSSVKAP 128

KY484518.Mandailing Natal-290.Indonesia SLKSTGRLSGMSSVKAP 128

KY562848.Bogor-43.Indonesia SLKSTGRLSGMSSVKAP 128

KY484511.Tangerang-38.Indonesia SPKNIGRLSGMSSVKAP 128

KY484513.Lombok Timur-160.Indonesia SPKNIGRLSGMSSVKAP 128

KY484515.Manggarai Timur-192.Indonesia SPKNIGRLSGMSSVKAP 128

KY484517.Tabalong-239.Indonesia SPKNIGRLSGMSSVKAP 129

KY484514.Kupang-181.Indonesia SPKNIGRISGMSSVKAP 129

KY484516.Malaka-209.Indonesia SPKNIGRLSGMSSVKAP 129

* *. * :*********

**Additional file 2: D.** Alignment of partial nucleotide sequences of *B. bigemina* rap-1a gene among Indonesian isolates. Nucleotide substitutions were indicated green background. The alignment was inferred by CLUSTAL O(1.2.4) multiple sequence alignment.

KY484521.Jombang-480.Indonesia AGCTTGCTTTCACAACTCGCCTGTTCGGTTTCGGTATCAAGAAGGCGTTGAAGCGTTTGG 60

KY484510.Karawang-6.Indonesia AGCTTGCTTTCACAACTCGCCTGTTCGGTTTCCGTATCCAGAAGGCGTTGAAGCGTTTGG 60

KY484520.Lamongan-448.Indonesia AGCTTGCTTTCACAACTCGCCTGTTCGGTTTCGGTATCCAGAAGGCGTTGAAGCGTTTGG 60

KY484512.Padang Mangateh-84.Indonesia AGCTTGCTTTCACAACTCGCCTGTTCGGTTTCGGTATCCAGAAGGCGTTGAAGCGTTTGG 60

KY484519.Bulukumba-366.Indonesia AGCTTGCTTTCACAACTCGCCTGTTCGGTTTCGGTATCCAGAAGGCGTTGAAGCGTTTGG 60

KY484518.Mandailing Natal-290.Indonesia AGCTTGCTTTCACAACTCGCCTGTTCGGTTTCGGTATCCAGAAGGCGTTGAAGCGTTTGG 60

KY562848.Bogor-43.Indonesia AGCTTGCTTTCACAACTCGCCTGTTCGGTTTCGGTATCCAGAAGGCGTTGAAGCGTTTGG 60

KY484511.Tangerang-38.Indonesia AGCTTGCTTTCACAACTCGCCTGTTCGGTTTCGGTATCCAGAGGGCGTTGAAGCGTTTGG 60

KY484513.Lombok Timur-160.Indonesia AGCTTGCTTTCACAACTCGCCTGTTCGGTTTCGGTATCCAGAGGGCGTTGAAGCGTTTGG 60

KY484515.Manggarai Timur-192.Indonesia AGCTTGCTTTCACAACTCGCCTGTTCGGTTTCGGTATCCAGAGGGCGTTGAAGCGTTTGG 60

KY484517.Tabalong-239.Indonesia AGCTTGCTTTCACAACTCGCCTGTTCGGTTTCGGTATCCAGAGGGCGTTGAAGCGTTTGG 60

KY484514.Kupang-181.Indonesia AGCTTGCTTTCACAACTCGCCTGTTCGGTTTCGGTATCCAGAGGGCGTTGAAGCGTTTGG 60

KY484516.Malaka-209.Indonesia AGCTTGCTTTCACAACTCGCCTGTTCGATATCGGTATCCAGAGGGCGTTGAAGCGTTTGG 60

*************************** * ** ***** *** *****************

KY484521.Jombang-480.Indonesia TTAGGAGCAACCTTCCCGTTGACCTTGGAACCCACCCTGAGGCCACCATCCGCGAAATAG 120

KY484510.Karawang-6.Indonesia TTAGGAGCAACCTTCCCGTTGACCTTGGAACCCACCCTGAGGCCACCATCCGCGAAATAG 120

KY484520.Lamongan-448.Indonesia TTAGGAGCAACCTTCCCGTTGACCTTGGAACCCACCCTGAGGCCACCATCCGCGAAATAG 120

KY484512.Padang Mangateh-84.Indonesia TTAGGAGCAACCTTCCCGTTGACCTTGGAACCCACCCTGAGGCCACCATCCGCGAAATAG 120

KY484519.Bulukumba-366.Indonesia TTAGGAGCAACCTTCCCGTTGACCTTGGAACCCACCCTGAGGCCACCATCCGCGAAATAG 120

KY484518.Mandailing Natal-290.Indonesia TTAGGAGCAACCTTCCCGTTGACCTTGGAACCCACCCTGAGGCCACCATCCGCGAAATAG 120

KY562848.Bogor-43.Indonesia TTAGGAGCAACCTTCCCGTTGACCTTGGAACCCACCCTGAGGCCACCATCCGCGAAATAG 120

KY484511.Tangerang-38.Indonesia TTAGGAGCAACCTTCCCGTTGACCTTGGAACCCACCCTGAGGCCACCATCCGCGAGATAG 120

KY484513.Lombok Timur-160.Indonesia TTAGGAGCAACCTTCCCGTTGACCTTGGAACCCACCCTGAGGCCACCATCCGCGAGATAG 120

KY484515.Manggarai Timur-192.Indonesia TTAGGAGCAACCTTCCCGTTGACCTTGGAACCCACCCTGAGGCCACCATCCGCGAGATAG 120

KY484517.Tabalong-239.Indonesia TTAGGAGCAACCTTCCCGTTGACCTTGGAACCCACCCTGAGGCCACCATCCGCGAGATAG 120

KY484514.Kupang-181.Indonesia TTAGGAGCAACCTTCCCGTTGACCTTGGAACCCACCCTGAGGCCACCATCCGGGAGATAG 120

KY484516.Malaka-209.Indonesia TTAGGAGCAACCTTCCCGTTGACCTTGGAACCCACCCTGAGGCCACCATCCGGGAGATAG 120

**************************************************** ** ****

KY484521.Jombang-480.Indonesia CTAGCGGCTACGGCGAGTACATGATGACCCAGGTGCCTGCGATGACCTCGTTCGCTGAGC 180

KY484510.Karawang-6.Indonesia CTAGCGGCTACGGCGAGTACATGATGACCCAGGTGCCTGCGATGACCTCGTTCGCTGAGC 180

KY484520.Lamongan-448.Indonesia CTAGCGGCTACGGCGAGTACATGATGACCCAGGTGCCTGCGATGACCTCGTTCGCTGAGC 180

KY484512.Padang Mangateh-84.Indonesia CTAGCGGCTACGGCGAGTACATGATGACCCAGGTGCCTGCGATGACCTCGTTCGCTGAGC 180

KY484519.Bulukumba-366.Indonesia CTAGCGGCTACGGCGAGTACATGATGACCCAGGTGCCTGCGATGACCTCGTTCGCTGAGC 180

KY484518.Mandailing Natal-290.Indonesia CTAGCGGCTACGGCGAGTACATGATGACCCAGGTGCCTGCGATGACCTCGTTCGCTGAGC 180

KY562848.Bogor-43.Indonesia CTAGCGGCTACGGCGAGTACATGATGACCCAGGTGCCTGCGATGACCTCGTTCGCTGAGC 180

KY484511.Tangerang-38.Indonesia CTAGCGGCTACGGCGAATACATGATGACCCAGGTGCCTGCGATGACCTCGTTCGCTGAGC 180

KY484513.Lombok Timur-160.Indonesia CTAGCGGCTACGGCGAATACATGATGACCCAGGTGCCTGCGATGACCTCGTTCGCTGAGC 180

KY484515.Manggarai Timur-192.Indonesia CTAGCGGCTACGGCGAATACATGATGACCCAGGTGCCTGCGATGACCTCGTTCGCTGAGC 180

KY484517.Tabalong-239.Indonesia CTAGCGGCTACGGCGAATACATGATGACCCAGGTGCCTGCGATGACCTCGTTCGCTGAGC 180

KY484514.Kupang-181.Indonesia ATAGCGGTTACGGCGAATACATGATGACCCAGGTGCCTGCGATGACCTCGTTCGCTGAGC 180

KY484516.Malaka-209.Indonesia ATAGCGGTTACGGCGAATACATGATGACCCAGGTGCCTGCGATGACCTCGTTCGCTGAGC 180

****** ******** *******************************************

KY484521.Jombang-480.Indonesia GTTTCTCCAAGATGGCTACTAAGACTCTGTTGGTTACCGTCAGCGACTACGTCCATTTGC 240

KY484510.Karawang-6.Indonesia GTTTCTCCAAGATGGCTACTAAGACTCTGTTGGTTACCGTCAGCGACTACGTCCATTTGC 240

KY484520.Lamongan-448.Indonesia GTTTCTCCAAGATGGCTACTAAGACTCTGTTGGTTACCGTCAGCGACTACGTCCATTTGC 240

KY484512.Padang Mangateh-84.Indonesia GTTTCTCCAAGATGGCTACTAAGACTCTGTTGGTTACCGTCAGCGACTACGTCCATTTGC 240

KY484519.Bulukumba-366.Indonesia GTTTCTCCAAGATGGCTACTAAGACTCTGTTGGTTACCGTCAGCGACTACGTCCATTTGC 240

KY484518.Mandailing Natal-290.Indonesia GTTTCTCCAAGATGGCTACTAAGACTCTGTTGGTTACCGTCAGCGACTACGTCCATTTGC 240

KY562848.Bogor-43.Indonesia GTTTCTCCAAGATGGCTACTAAGACTCTGTTGGTTACCGTCAGCGACTACGTCCATTTGC 240

KY484511.Tangerang-38.Indonesia GTTTCTCCAAGATGGCTACTAAGACTCTGTTGGTTACCGTCAGCGACTACGTCCGGTTGC 240

KY484513.Lombok Timur-160.Indonesia GTTTCTCCAAGATGGCTACTAAGACTCTGTTGGTTACCGTCAGCGACTACGTCCGGTTGC 240

KY484515.Manggarai Timur-192.Indonesia GTTTCTCCAAGATGGCTACTAAGACTCTGTTGGTTACCGTCAGCGACTACGTCCGGTTGC 240

KY484517.Tabalong-239.Indonesia GTTTCTCCAAGATGGCTACTAAGACTCTGTTGGTTACCGTCAGCGGCTACGTCCGGTTGC 240

KY484514.Kupang-181.Indonesia GTTTATCCAAGATGGGTACTAAGACTCTGTTGGTTACCGTCAGCGACTACGTCCGGTTGC 240

KY484516.Malaka-209.Indonesia GTTTCTCCAAGATGGCTACTAAGACTCTGTTGGTTACCGTCAGCGACTACGTCCGGTTGC 240

**** ********** ***************************** ******** ****

KY484521.Jombang-480.Indonesia CCGCGTACAAGAGGTGGTACAGGAAGTTCAAGGAGTTCATTGTGAGCTTCTTTACTGACC 300

KY484510.Karawang-6.Indonesia CCGCGTACAAGAGGTGGTACAGGAAGTTCAAGGAGTTCATTGTGAACTTCTTTACTGACC 300

KY484520.Lamongan-448.Indonesia CCGCGTACAAGAGGTGGTACAGGAAGTTCAAGGAGTTCATTGTGAACTTCTTTACTGACC 300

KY484512.Padang Mangateh-84.Indonesia CCGCGTACAAGAGGTGGTACAGGAAGTTCAAGGAGTTCATTGTGAACTTCTTTACTGACC 300

KY484519.Bulukumba-366.Indonesia CCGCGTACAAGAGGTGGTACAGGAAGTTCAAGGAGTTCATTGTGAACTTCTTTACTGACC 300

KY484518.Mandailing Natal-290.Indonesia CCGCGTACAAGAGGTGGTACAGGAAGTTCAAGGAGTTCATTGTGAACTTCTTTACTGACC 300

KY562848.Bogor-43.Indonesia CCGCGTACAAGAGGTGGTACAGGAAGTTCAAGGAGTTCATTGTGAACTTCTTTACTGACC 300

KY484511.Tangerang-38.Indonesia CCGCGTACAAGAGGTGGTACAGGAAGTTCAAGGAGTTCATTGTGACCTTCTTTACCGACC 300

KY484513.Lombok Timur-160.Indonesia CCGCGTACAAGAGGTGGTACAGGAAGTTCAAGGAGTTCATTGTGACCTTCTTTACCGACC 300

KY484515.Manggarai Timur-192.Indonesia CCGCGTACAAGAGGTGGTACAGGAAGTTCAAGGAGTTCATTGTGACCTTCTTTACCGACC 300

KY484517.Tabalong-239.Indonesia CCGCGTACAAGAGGTGGTACAGGAAGTTCAAGGAGTTCATTGTGACCTTCTTTACCGACC 300

KY484514.Kupang-181.Indonesia CCGCGTACAAGAGGTGGTACAGGAAGTTCAAGGAGTTCATTGTGACCTTCTTTACCGACC 300

KY484516.Malaka-209.Indonesia CCGCGTACAAGAGGTGGTACAGGAAGTTCAAGGAGTTCATTGTGACCTTCTTTACCGACC 300

********************************************* ********* ****

KY484521.Jombang-480.Indonesia CTGCCAAGTTGATTATGAAGCACGTCTCTCAGCCTGTAAAGACTGCCTACACAAAGCTGG 360

KY484510.Karawang-6.Indonesia CTGCCAAGTTGATTATGAAGCACGTCTCTCAGCCTGTAAAGACTGCCTACACAAAGCTGG 360

KY484520.Lamongan-448.Indonesia CTGCCAAGTTGATTATGAAGCACGTCTCTCAGCCTGTAAAGACTGCCTACACAAAGCTGG 360

KY484512.Padang Mangateh-84.Indonesia CTGCCAAGTTGATTATGAAGCACGTCTCTCAGCCTGTAAAGACTGCCTACACAAAGCTGG 360

KY484519.Bulukumba-366.Indonesia CTGCCAAGTTGATTATGAAGCACGTCTCTCAGCCTGTAAAGACTGCCTACACAAAGCTGG 360

KY484518.Mandailing Natal-290.Indonesia CTGCCAAGTTGATTATGAAGCACGTCTCTCAGCCTGTAAAGACTGCCTACACAAAGCTGG 360

KY562848.Bogor-43.Indonesia CTGCCAAGTTGATTATGAAGCACGTCTCTCAGCCTGTAAAGACTGCCTACACAAAGCTGG 360

KY484511.Tangerang-38.Indonesia CTGCCAAGTTGATTTTGAAGCACGTCTCTCAGCCTGTGAAGACTGCGTACACAAAGCTGG 360

KY484513.Lombok Timur-160.Indonesia CTGCCAAGTTGATTTTGAAGCACGTCTCTCAGCCTGTGAAGACTGCGTACACAAAGCTGG 360

KY484515.Manggarai Timur-192.Indonesia CTGCCAAGTTGATTTTGAAGCACGTCTCTCAGCCTGTGAAGACTGCGTACACAAAGCTGG 360

KY484517.Tabalong-239.Indonesia CTGCCAAGTTGATTTCGAAGCACGTCTCTCAGCCTGTGAGGACTGCGTACACAAAGCTGG 360

KY484514.Kupang-181.Indonesia CTGCCAAGTTGATTTTGAAGCACGTCTCTCAGCCTGGGAAGACTGCGTACACAAAGCTGG 360

KY484516.Malaka-209.Indonesia CTGCCAAGTTGATTTTGAAGCACGTCTCTCAGCCTGGGAAGACTGCGTACACAAAGCTGG 360

************** ******************** * ****** *************

KY484521.Jombang-480.Indonesia TCCCTGAAGAGCACAGGCAGGCTATCAGGGATGTCGTCGGTCAAAGCACCAA 412

KY484510.Karawang-6.Indonesia TCCCTGAAGAGCACAGGCAGGCTATCAGGGATGTCGTCGGTCAAAGCACCAA 412

KY484520.Lamongan-448.Indonesia TCCCTGAAGAGCACAGGCAGGCTATCAGGGATGTCGTCGGTCAAAGCACCAA 412

KY484512.Padang Mangateh-84.Indonesia TCCCTGAAGAGCACAGGCATGCTATCAGGAATGTCGTCGGTCAAAGCACCAA 412

KY484519.Bulukumba-366.Indonesia TCCCTGAAGAGCGCAGGCAGGCTATCAGGAATGTCGTCGGTCAAAGCACCAA 412

KY484518.Mandailing Natal-290.Indonesia TCCCTGAAGAGCACAGGCAGGCTATCAGGAATGTCGTCGGTCAAAGCACCAA 412

KY562848.Bogor-43.Indonesia TCCCTGAAGAGCACAGGCAGGCTATCAGGAATGTCGTCGGTCAAAGCACCAA 412

KY484511.Tangerang-38.Indonesia TCCCCGAAGAACATAGGCAGGCTATCAGGAATGTCGTCGGTCAAAGCACCAA 412

KY484513.Lombok Timur-160.Indonesia TCCCCGAAGAACATAGGCAGGCTATCAGGAATGTCGTCGGTCAAAGCACCAA 412

KY484515.Manggarai Timur-192.Indonesia TCCCCGAAGAACATAGGCAGGCTATCAGGAATGTCGTCGGTCAAAGCACCAA 412

KY484517.Tabalong-239.Indonesia TCCCCGAAGAACATAGGCAGGCTATCAGGAATGTCGTCGGTCAAAGCACCAA 412

KY484514.Kupang-181.Indonesia TCCCCGAAGAACATAGGCAGGATATCAGGAATGTCGTCGGTCAAAGCACCAA 412

KY484516.Malaka-209.Indonesia TCCCCGAAGAACATAGGCAGGCTATCAGGAATGTCGTCGGTCAAAGCACCAA 412

**** ***** * ***** * ******* **********************

**Additional file 2. E.** Alignment of nucleotide sequences *B. bovis* ITS region. Alignments were inferred by CLUSTAL O (1.2.4) multiple sequence alignment [29].

MF664384.Tapanuli_Selatan_316.Indonesia -----------------CACCACCAGTGGAAGCACAGCTTCCACTCGGCACCTTCGGTGC 43

MF664385.Jombang_496.Indonesia CACCACCAGTGGAAGCACAGCTTCCACCTGAGCACTTTGTGCTCACGGCACCTCCGGTGC 60

MF664377.Karawang_17.Indonesia CACCACCAGTGGAAGCACAGCTTCCACCTGAGCACTTTGTGCTCACGGCACCTCCGGTGC 60

MF664383.Mandailing_Natal_293.Indonesia CACCACCAGTGGAAGCACAGCTTCCACCTGAGCACTTCGTGCTCACGGCACCTTCGGTGC 60

MF664380.Kupang_167.Indonesia CACCACCAGTGGAAGCACAGCTTCCACCCAACGAGTACT-----------TTGTACTCGC 49

MF664381.Manggarai_Timur_190.Indonesia CACCACCAGTGGAAGCACAGCTTCCA---------------------------------C 27

MF664382.Malaka_210.Indonesia CACCACCAGTGGAAGCACAGCTTCCACCTGAG---CACT-----------CCGTGCTCAC 46

MF664378.Padang_Mangateh_109.Indonesia CACCACCAGTGGAAGCACAGCTTCCACCTGAG---CAC------------TCCGTGCTCA 45

MF664379.Dompu_146.Indonesia CACCACCAGTGGAAGCACAGCTTCCACCTGAG---CACT-----------CTCGTGCTCA 46

** * *

MF664384.Tapanuli_Selatan_316.Indonesia CACTGATCGCCTTGCGGCGATCTGGCAACGCCGGCTACCCTAGTAGCCGGTTGGGGCTCC 103

MF664385.Jombang_496.Indonesia CACTGATCGCCTTGCGGCGATTTGGCAACGCCGGCTACCCTAGTAGCCGGTTGGGGCTTT 120

MF664377.Karawang_17.Indonesia C----------------ACTACTAGCAACGCCGGCTACCCTAGTAGCCGGTTGGGGCTCC 104

MF664383.Mandailing_Natal_293.Indonesia C----------------ACTACTAGCAACGCCGGCTACCCTAGTAGCCGGTTGGGGCTCC 104

MF664380.Kupang_167.Indonesia GAGCACTTCGTGCTCGGCCATCTGGCAACGCCGGCTACCCTAGTAGCCGGTTGGGGCTTT 109

MF664381.Manggarai_Timur_190.Indonesia CCGGCACCTCCGGTGCCACTACTAGCAACGCCGGCTACCCTAGTAGCCGGTTGGGGCTCC 87

MF664382.Malaka_210.Indonesia ---CCGATCGCCTTTGGCGATCTGGCAACGCCGGCTACCCTAGTAGCCGGTTGGGGCTCC 103

MF664378.Padang_Mangateh_109.Indonesia CTGGCACCTCCGGTGCCACTACTAGCAACGCCGGCTACCCTAGTAGCCGGTTGGGGCTCC 105

MF664379.Dompu_146.Indonesia CTGGCACCTCCGGTGCCACTACTAGCAACGCCGGCTACCCTAGTAGCCGGTTGGGGCTCC 106

* **********************************

MF664384.Tapanuli_Selatan_316.Indonesia GCCCCCGTTGCTCCCCACCCCGAGGGCCGTGACTGCCACGACCCGGGTTAAGCTCGCCTC 163

MF664385.Jombang_496.Indonesia GCCCCCGTTGCTCCCCACCCCGAGGGCCGTGACTGCCACGACCCGGGTTAAGCTCGCCTC 180

MF664377.Karawang_17.Indonesia GCCCCCGTTGCTCCCCACCCCGAGGGCCGTGACTGCCACGACCCGGGTTAAGCTCGCCCC 164

MF664383.Mandailing_Natal_293.Indonesia GCCCCCGTTGCTCCCCACCCCGAGGGCCGTGACTGCCACGACCCGGGTTAAGCTCGCCTC 164

MF664380.Kupang_167.Indonesia GCCCCCGTTGCTCCCCACCCCGAGGGCCGTGACTGCCACGACCCGGGTTAAGCTCGCCTC 169

MF664381.Manggarai_Timur_190.Indonesia GCCCCCGTTGCTCCCCACCCCGAGGGCCGTGACTGCCACGACCCGGGTTAAGCTCGCCTC 147

MF664382.Malaka_210.Indonesia GCCCCCGTTGCTCCCCACCCCGAGGGCCGTGACTGCCACGACCCGGGTTAGGCTCGCCCC 163

MF664378.Padang_Mangateh_109.Indonesia GCCCCCGTTGCTCCCCACCCCGAGGGCCGTGACTGCCACGACCCGGGTTAAGCTCGCCCC 165

MF664379.Dompu_146.Indonesia GCCCCCGTTGCTCCCCACCCCGAGGGCCGTGACTGCCACGACCCGGGTTAAGCTCGCCCC 166

************************************************** ******* *

MF664384.Tapanuli_Selatan_316.Indonesia GGCGAGATGCACCCCCTTTTTGGG-GGTGCTTTCCAGCCCTTTAGGGCTGGCACAACTAC 222

MF664385.Jombang_496.Indonesia GGCGAGATGCACCCCCTTTTTGGG-GGTGCCCTCCAGCCCTTTAGGGGTGGCACAATTAC 239

MF664377.Karawang_17.Indonesia GGCGAGATGCACCCCCTTTTTTGGGGGTGCCTTCCAGCCCTTTAGGGCTGGCACAATTAC 224

MF664383.Mandailing_Natal_293.Indonesia GGCGAGATGCACCCCCAAAAGG--GGGTGCCTACCAGCCCTTTAGGGCTGGCACAACCAC 222

MF664380.Kupang_167.Indonesia GGCGAGATGCACCCCCTTGGGGGT----GCCCTCCAGCCCTCTAGGGCTGGCACCAGCAC 225

MF664381.Manggarai_Timur_190.Indonesia GGCGAGATGCACCCCCTTGGGGGT----GCCTTCCAGCCCTCCAGGGCTGGCATAACTAC 203

MF664382.Malaka_210.Indonesia GGCGAGATGCACCCCCTTGGGGGT----GCCCTCCAGCCCTCTAGGGCTGGCATAACCAC 219

MF664378.Padang_Mangateh_109.Indonesia GGCGAGATGCACCCCCTCGGGGGT----GCCTTCCAGCCCGCTAGGGCTGGCATAACTAC 221

MF664379.Dompu_146.Indonesia GGCGAGATGCACCCCCTCGGGGGT----GCCTTCCAGCCCGCTAGGGCTGGCATAACTAC 222

**************** ** ******* **** ***** * **

MF664384.Tapanuli_Selatan_316.Indonesia ACC---ACACTCAATACACTCTAAACTCCCAGCGATGGATGCCTCGGCTCGCGCCTCGAT 279

MF664385.Jombang_496.Indonesia CCC---ACCCCACCA-TCACTACAACTCCCAGCGATGGATGCCTCGGCTCGCGCCTCGAT 295

MF664377.Karawang_17.Indonesia --A--CCACCACACAATCACTACAACTCCCAGCGATGGATGCCTCGGCTCGCGCCTCGAT 280

MF664383.Mandailing_Natal_293.Indonesia TCA--CTAGACCATCACTACCTAAACTCCCAGCGATGGATGCCTCGGCTCGCGCCTCGAT 280

MF664380.Kupang_167.Indonesia AG---CACACTGACTACACTCTAAACTCCCAGCGATGGATGCCTCGGCTCGCGCCTCGAT 282

MF664381.Manggarai_Timur_190.Indonesia TCAC-TAGACCAATCACTACCTAAACTCCCAGCGATGGATGCCTCGGCTCGCGCCTCGAT 262

MF664382.Malaka_210.Indonesia CACAGCACTGACTACACTATC-CAACTCCCAGCGATGGATGCCTCGGCTCGCGCCTCGAT 278

MF664378.Padang_Mangateh_109.Indonesia CACAGCACTGACTACACTACCTCAACTCCCAGCGATGGATGCCTCGGCTCGCGCCTCGAT 281

MF664379.Dompu_146.Indonesia CACAGCACTGACTACACTACCTCAACTCCCAGCGATGGATGCCTCGGCTCGCGCCTCGAT 282

*************************************

MF664384.Tapanuli_Selatan_316.Indonesia GAAGGACGCAGCAAAGTGCGATATCCAGCATGATTTGCAACTTCTTGCGATTGCTAGACC 339

MF664385.Jombang_496.Indonesia GAAGGACGCAGCAAAGTGCGATATCCAGCATGATTTGCAACTTCTTGCGATTGCTAGACC 355

MF664377.Karawang_17.Indonesia GAAGGACGCAGCAAAGTGCGATATCCAGCATGATTTGCAACTTCTTGCGATTGCTAGACC 340

MF664383.Mandailing_Natal_293.Indonesia GAAGGACGCAGCAAAGTGCGATATCCAGCATGATTTGCAACTTCTTGCGATTGCTAGACC 340

MF664380.Kupang_167.Indonesia GAAGGACGCAGCAAAGTGCGATATCCAGCATGATTTGCAACTTCTTGCGATTGCTAGACC 342

MF664381.Manggarai_Timur_190.Indonesia GAAGGACGCAGCAAAGTGCGATATCCAGCATGATTTGCAACTTCTTGCGATTGCTAGACC 322

MF664382.Malaka_210.Indonesia GAAGGACGCAGCAAAGTGCGATATCCAGCATGATTTGCAACTTCTTGCGATTGCTAGACC 338

MF664378.Padang_Mangateh_109.Indonesia GAAGGACGCAGCAAAGTGCGATATCCAGCATGATTTGCAACTTCTTGCGATTGCTAGACC 341

MF664379.Dompu_146.Indonesia GAAGGACGCAGCAAAGTGCGATATCCAGCATGATTTGCAACTTCTTGCGATTGCTAGACC 342

************************************************************

MF664384.Tapanuli_Selatan_316.Indonesia TCTGAACGTAACCAACACACGCTTGTACGTCCATCTCAGTGAATTTCCAGTATGGCACAA 399

MF664385.Jombang_496.Indonesia TCTGAACGTAACCCACACACTCTTGTACGTCCATCTCAGTGAATTTTCCATATGGGACAA 415

MF664377.Karawang_17.Indonesia TCTGAACGTAACCAACACACTCTTGTACGTCCATCTCAGTGAATTTCCAGTATGGTGTGA 400

MF664383.Mandailing_Natal_293.Indonesia TCTGAACGTAACCAACACACTTATGTACGTCCATCTCAGTGAATTCCCCATATGGTGTGA 400

MF664380.Kupang_167.Indonesia TCTGAACGTAACCAACACACGCTTGTACGTCCATCTCAGTGAATCTTCCATATGGTGTGA 402

MF664381.Manggarai_Timur_190.Indonesia TCTGAACGTAACCAACACACGCTTGTACGTCCATCTCAGTGAATTTTCAGTATGGTGTGA 382

MF664382.Malaka_210.Indonesia TCTGAACGTAACCAACACACTCTTGTACGTCCATCTCAGTGAATTTTTCAGTAATGGCAC 398

MF664378.Padang_Mangateh_109.Indonesia TCTGAACGTAACCAACACACGCTTGTACGTCCATCTCAGTGAATTTTCAGTATGGCACA- 400

MF664379.Dompu_146.Indonesia TCTGAACGTAACCAACACACGCTTGTACGTCCATCTCAGTGAATTTTCAGTATGGCACA- 401

************* ****** *********************

MF664384.Tapanuli_Selatan_316.Indonesia TGCCGCAAT--------------------GGCAGGGCGTTAGCCCTGCTCTTC-C--TAA 436

MF664385.Jombang_496.Indonesia TGCCGCCAT--------------------GGGAGGGGGATAGCCCTGCTCCTTCA--CGA 453

MF664377.Karawang_17.Indonesia CACAGCGCACCAGTGTGTTGCACCGCCATGGCAGGGCGTCAGCCCTGCTCCTACTAAAG- 459

MF664383.Mandailing_Natal_293.Indonesia CACACCACCA----GTGTTGCACCGCCATGGCAGGGCGTTAGCCCTGCTCCTTACTAAAG 456

MF664380.Kupang_167.Indonesia CACACCACCAGTGTGTTGCACCGCC--CTGGCGGTG--CCTCTACAC--CGCTTTTCCCA 456

MF664381.Manggarai_Timur_190.Indonesia CACACCACCAGTGTGTTGCACCGCC--CTGGCGGTG--CCTCTACAC--CGCTATTCCCA 436

MF664382.Malaka_210.Indonesia AATGCCGTTAATGGGCAGGG---CG--T-----------TAGCCCTG--CTTTTCCAAAG 440

MF664378.Padang_Mangateh_109.Indonesia -ATGCCGT--AATGGCAGGG---CG--T-----------TAGCCCTG--CTCC--TTCAC 437

MF664379.Dompu_146.Indonesia -ATGCCGT--AATGGCAGGG---CG--T-----------TAGCCCTG--CTCC--TTCAC 438

* * *

MF664384.Tapanuli_Selatan_316.Indonesia AGGCACACTGTGACCCCGACACGATAGATTTATAGTGTCCATGGGGCACAA 487

MF664385.Jombang_496.Indonesia GGACACACTGTGACCCCGACACGATAGATTTATAGTGTCCATGGGGCACAA 504

MF664377.Karawang_17.Indonesia --GCACACTGTGACCCCGACACGATAGATTTATAGTGTCCATGGGGCACAA 508

MF664383.Mandailing_Natal_293.Indonesia GACACTACTGTGACCCCGACACGATAGATTTATAGTGTCCATGGGGCACAA 507

MF664380.Kupang_167.Indonesia GAGGACACTCTGCTCCCGACACGATAGATTTACAGTGTCCATGGGGCACAA 507

MF664381.Manggarai_Timur_190.Indonesia GAGGACACTCCGCTCCCGACACGATAGATTCACAGTGTCCATGGGGCACAA 487

MF664382.Malaka_210.Indonesia GAAAGCACTCCGCTCCCGACACGATAGATTTACAGTGTCCATGGGGCACAA 491

MF664378.Padang_Mangateh_109.Indonesia GAGGACACTCCGCTCCCGACACGATAGATTTACAGTGTCCATGGGGCACAA 488

MF664379.Dompu_146.Indonesia GAGGACACTCCGCTCCCGACACGATAGATTTACAGTGTCCATGGGGCACAA 489

*** * **************** * ******************

**Additional file 2. F.** Alignment of nucleotide sequences *B. bigemina* ITS region. Alignments were inferred by CLUSTAL O (1.2.4) multiple sequence alignment [29].

MF664392.Mandailing_Natal-290.Indonesia AGTGGTCGGGACTCGTCCGGCGCGTTTTTCTAAAATGCGTTCCCTTTTGGGCTTCCACTG 60

MF664387.Lombok_Timur-160.Indonesia AGTGGTCGGGACTCGTCCGG---CGCGTCTTAGGATGCGTTCC-CTTTGGGCTTCCACTG 56

MF664390.Malaka-209.Indonesia AGTGGTCGGGACTCGTCCGG---CGCGTCCTTGGATGCGTTCCCTTTTGGGCTTCCACTG 57

MF664388.Kupang-181.Indonesia AGTGGTCGGGACTCGTCCGG---CGCGTCTTAGGATGCGTTCCC-TTTGGGCTTCCACTG 56

MF664386.Dompu-140.Indonesia AGTGGTCGGGACTCGTCCGG---CGCGTCTTAGGATGCGTTCCCTTTTGGGCTTCCACTG 57

MF664389.Manggarai_Timur-192.Indonesia AGTGGTCGGGACTCGTCCGG---CGCGTCCTTGGATGCGTTCCCTTTTGGGCTTCCACTG 57

MF664391.Tabalong-279.Indonesia AGTGGTCGGGACTCGTCCGG---CGCGTCTTAGGATGCGTTCC-CTTTGGGCTTCCACTG 56

MF664393.Tapanuli_Selatan-317.Indonesia AGTGGTCGGGACTCGTCCGG---CGCGTCTTAGGATGCGTTCC-CTTTGGGCCTCCACTG 56

******************** * * ********* ******* *******

MF664392.Mandailing_Natal-290.Indonesia CTTCTTGCGCCAGCAACGCGCCCTTGCTGTTTGCTCGGGCTGCCCGGCGTTATCGCTGGG 120

MF664387.Lombok_Timur-160.Indonesia CGTCTTGCGCCAGCAACGCGCCCTTGCTTTTTGCTCGGGCTGCCCGGCGATTTCGCTGGG 116

MF664390.Malaka-209.Indonesia CGTCTTGCGCCAGCAACGCGCCCTTGCTTTTTGCTCGGGCTGCCCGGCGATTTCGCTGGG 117

MF664388.Kupang-181.Indonesia CGTCTTGCGCCAGCAACGCGCCCTTGCTTTTTGCTCGGGCTGCCCGGCGTTATCGCTGGG 116

MF664386.Dompu-140.Indonesia CGTCTTGCGCCAGCAACGCGCCCTTGCTGTTTGCTCGGGCTGCCCGGCGTTATCGCTGGG 117

MF664389.Manggarai_Timur-192.Indonesia CGTCTTGCGCCAGCAACGCGCCCTTGCTTTTTGCTCGGGCTGCCCGGCGATTTCGCTGGG 117

MF664391.Tabalong-279.Indonesia CTTCTTGCGCCAGCAACGCGCCCTTGCCTTTTGCTCGGGCTGCCCGGCGTTTTCGCTGGG 116

MF664393.Tapanuli_Selatan-317.Indonesia CTTCTTGCGCCAGCAACGCGCCCTTGCCTTTTGCTCGGGCTGCCCGGCGATTTCGCTGGG 116

* ************************* ******************** * ********

MF664392.Mandailing_Natal-290.Indonesia GCGTTGCCTCCTCCCACCCCGTGCTCGCGTTGTCGTCGCTCTTGCAGCGTGCTCTGCGAG 180

MF664387.Lombok_Timur-160.Indonesia GCGTTGCCTCCTCCCACCCCGTGCTCGCGTTGTCGTCGTTTTTGCAGCGTGCTCTGCGAG 176

MF664390.Malaka-209.Indonesia GCGTTGCCTCCTCCCACCCCGTGCTCGCGTTGTCGTCGCTATTGCAGCGTGCTCTGCGAG 177

MF664388.Kupang-181.Indonesia GCGTTGCCTCCTCCCACCCCGTGCTCGCGTTGTCGTCGCTATTGCAGCGTGCTCTGCGAG 176

MF664386.Dompu-140.Indonesia GCGTTGCCTCCTCCCACCCCTTGCTCGCGTTGTCGTCGCTATTGCAGCGTGCTCTGCGAG 177

MF664389.Manggarai_Timur-192.Indonesia GCGTTGCCTCCTCCCACCCCTTGCTCGCGTTGTCGTCGCTATTGCAGCGTGCTCTGCGAG 177

MF664391.Tabalong-279.Indonesia GCGTTGCCTCCTCCCACCCCTTGCTCGCGTTGTCGTCGCTCTTGCAGCGTGCTCTGCGAG 176

MF664393.Tapanuli_Selatan-317.Indonesia GCGTTGCCTCCTCCCACCCCGCGCTCGCGTTGTCGTCGCTCTTGCAGCGTGCTCTGCGAG 176

******************** **************** * *******************

MF664392.Mandailing_Natal-290.Indonesia CGGGTTCCGCCCCCGTCCGTCGCTAGCATGTCGCGGTTTATTGCCGTGTCGTCTGGCAGC 240

MF664387.Lombok_Timur-160.Indonesia CGGGTTCCGCCCCCGTCCGTCGCTGTGATGTCGCGGTTTCGTGCCGCGTGCTCTGGCGTC 236

MF664390.Malaka-209.Indonesia CGGGTTCCGCCCCCGTCCGTCGCTATGATGTCGCGGCTTCGTGCCGTGTGGTCTGGCGTC 237

MF664388.Kupang-181.Indonesia CGGGTTCCGCCCCCGTCCGTCGCTAGTATGTCGCGGCTTCGTGCCGTGTGGTCTGGCGTC 236

MF664386.Dompu-140.Indonesia CGGGTTCCGCCCCCGTCCGTCGCTAGTATGTCGCGGTTTCGTGCCGTGTCGCCTGGCGTC 237

MF664389.Manggarai_Timur-192.Indonesia CGGGTTCCGCCCCCGTCCGTCGCTAGTATGTCGCGGTTTCGTGCCGTGTCGCCTGGCGTC 237

MF664391.Tabalong-279.Indonesia CGGGTTCCGCCCCCGTCCGTCGCTAGCATGTCGCGGTTCATTGCCGTGTCGTCTGGCATC 236

MF664393.Tapanuli_Selatan-317.Indonesia CGGGTTCCGCCCCCGTCCGTCGCTAGCATGTCGCGGTTTATTGCCGTGTCGTCTGGCAGC 236

************************ ********* * ***** ** ***** *

MF664392.Mandailing_Natal-290.Indonesia GGTCGGGGGATGTCGCTGCGCCGTGTGTGCGAGCGACCGCCGTCACTCAGCGTTGCTGTG 300

MF664387.Lombok_Timur-160.Indonesia GGTCGGGGGATGTCGCTGCGCCGTTTGTGCGAGCGAGCGCCGTGCCTCAGCGTTGCTGTG 296

MF664390.Malaka-209.Indonesia GGTCGGGGGATGTCGCTGCGCCGTTTGTGCGAGCGAGCGCCGTGCCTCAGCGTTGCTGTG 297

MF664388.Kupang-181.Indonesia GGTCGGGGGATGTCGCTGCGCCGTTTGTGCGAGCGAGCGCCGTGTGTCAGCGTTGCTGTG 296

MF664386.Dompu-140.Indonesia GGTCGGGGGATGTCGCTGCGCCGTTTGTGCGAGCGAGCGCCGTGTCTCAGCGTTGCTGTG 297

MF664389.Manggarai_Timur-192.Indonesia GGTCGGGGGATGTCGCTGCGCCGTTTGTGCGAGCGAGCGCCGTGTGTCAGCGTTGCTGTG 297

MF664391.Tabalong-279.Indonesia GGTCGGGGGATGTCGCTGCGCCGTTTGTGCGAGCGACCGCCGTCACTCAGCGTTGCTGTG 296

MF664393.Tapanuli_Selatan-317.Indonesia GGTCGGGGGATGTCGCTGCGCCGTTTGTGCGAGCGACCGCCGTGTCTCAGCGTTGCTGTG 296

************************ *********** ****** **************

MF664392.Mandailing_Natal-290.Indonesia CCTCGGCTGCCTTTTGGTTGTTGCAACTCCGCGCCT-CTGGCGTCTTTGTAAACTTTAAA 359

MF664387.Lombok_Timur-160.Indonesia TCTCGGCTGCCTTTTGGTTGTTGCAACTCCGCGCCTTTTGGCGTCTCTGTAAACTTTAAA 356

MF664390.Malaka-209.Indonesia TCTCGGCTGCCTTTTGGTTGTTGCAACTCCGCGCCTTTTGGCGTCTCTGTAAACTTTAAA 357

MF664388.Kupang-181.Indonesia TCTCGGCTGCCTTCTGGTTGTTGCAACTCCGCGCCTTTTGGCGTCTCTGTAAACTTTAAA 356

MF664386.Dompu-140.Indonesia TCTCGGCTGCCTTTTGGTTGTTGCAACTCCGCGCCTTTTGGCGTCTCTGTAAACTTTAAA 357

MF664389.Manggarai_Timur-192.Indonesia TCTCGGCTGCCTTTTGGTTGTTGCAACTCCGCGCCTTTTGGCGTCTCTGTAAACTTTAAA 357

MF664391.Tabalong-279.Indonesia CCTCGGCTGCCTTTTGGTTGTTGCAACTCCGCGCCT-CTGGCGTCTTTGTAAACTTTAAA 355

MF664393.Tapanuli_Selatan-317.Indonesia CCTCGGCTGCCTTTTGGTTGTTGCAACTCCGCGCCTTTTGGCGTCTTTGTAAACTTTAAA 356

************ ********************** ******** *************

MF664392.Mandailing_Natal-290.Indonesia CTTTCAGCGATGGATGTCTTGGCTCACACAACGATGAAGGACGCAGCGAATTGCGATACG 419

MF664387.Lombok_Timur-160.Indonesia CTTTCAGCGATGGATGTCTTGGCTCACACAACGATGAAGGACGCAGCGAATTGCGATACG 416

MF664390.Malaka-209.Indonesia CTTTCAGCGATGGATGTCTTGGCTCACACAACGATGAAGGACGCAGCGAATTGCGATACG 417

MF664388.Kupang-181.Indonesia CTTTCAGCGATGGATGTCTTGGCTCACACAACGATGAAGGACGCAGCGAATTGCGATACG 416

MF664386.Dompu-140.Indonesia CTTTCAGCGATGGATGTCTTGGCTCACACAACGATGAAGGACGCAGCGAATTGCGATACG 417

MF664389.Manggarai_Timur-192.Indonesia CTTTCAGCGATGGATGTCTTGGCTCACACAACGATGAAGGACGCAGCGAATTGCGATACG 417

MF664391.Tabalong-279.Indonesia CTTTCAGCGATGGATGTCTTGGCTCACACAACGATGAAGGACGCAGCGAATTGCGATACG 415

MF664393.Tapanuli_Selatan-317.Indonesia CTTTCAGCGATGGATGTCTTGGCTCACACAACGATGAAGGACGCAGCGAATTGCGATACG 416

************************************************************

MF664392.Mandailing_Natal-290.Indonesia CAGTATGACTTGCAGACTTCTGCGATTTACCAGACCTCTGAACG-TAACAAACACACCGC 478

MF664387.Lombok_Timur-160.Indonesia CAGTATGACTTGCAGACTTCTGCGATTTACCAGACCTCTGAACG-TAACAAACACACCGC 475

MF664390.Malaka-209.Indonesia CAGTATGACTTGCAGACTTCTGCGATTTACCAGACCTCTGA-ACGTAACAAACACACCGC 476

MF664388.Kupang-181.Indonesia CAGTATGACTTGCAGACTTCTGCGATTTACCAGACCTCTGAACG-TAACAAACACACCGC 475

MF664386.Dompu-140.Indonesia CAGTATGACTTGCAGACTTCTGCGATTTACCAGACCTCTGAACG-TAACAAACACACCGC 476

MF664389.Manggarai_Timur-192.Indonesia CAGTATGACTTGCAGACTTCTGCGATTTACCAGACCTCTGAACG-TAACAAACACACCGC 476

MF664391.Tabalong-279.Indonesia CAGTATGACTTGCAGACTTCTGCGATTTACCAGACCTCTGAACTGTAACAAACACACCGC 475

MF664393.Tapanuli_Selatan-317.Indonesia CAGTATGACTTGCAGACTTCTGCGATTTACCAGACCTCTGAACGTA-ACAAACACACCGC 475

***************************************** *************

MF664392.Mandailing_Natal-290.Indonesia CTCTGCTCGCACGCGGTACT 498

MF664387.Lombok_Timur-160.Indonesia CTCTGCTCGCACGCGGTACT 495

MF664390.Malaka-209.Indonesia CTCTGCTCGCACGCGGTACT 496

MF664388.Kupang-181.Indonesia CTCTGCTCGCACGCGGTACT 495

MF664386.Dompu-140.Indonesia CTCTGCTCGCACGCGGTACT 496

MF664389.Manggarai_Timur-192.Indonesia CTCTGCTCGCACGCGGTACT 496

MF664391.Tabalong-279.Indonesia CTCTGCTCGCACGCGGTACT 495

MF664393.Tapanuli_Selatan-317.Indonesia CTCTGCTCGCACGCGGTACT 495

********************
