# Supplementary material for: Molecular and serological detection of bovine babesiosis in Indonesia
Source: Parasit Vectors. 2017 Nov 6;10:550. doi: 10.1186/s13071-017-2502-0 (PMC5674684; doi:10.1186/s13071-017-2502-0)

Additional file 4: Figure S1 Phylogenetic tree of *Babesia bovis* sbp4 gene sequences. The maximum likelihood method based on the Kimura 2-parameter model with 1000 bootstrap replicates, available in MEGA ver.7, was used to determine the evolutionary history [30, 40]. All positions containing gaps and missing data were eliminated. Indonesian *B. bovis* sbp4 sequences are indicated by diamonds.

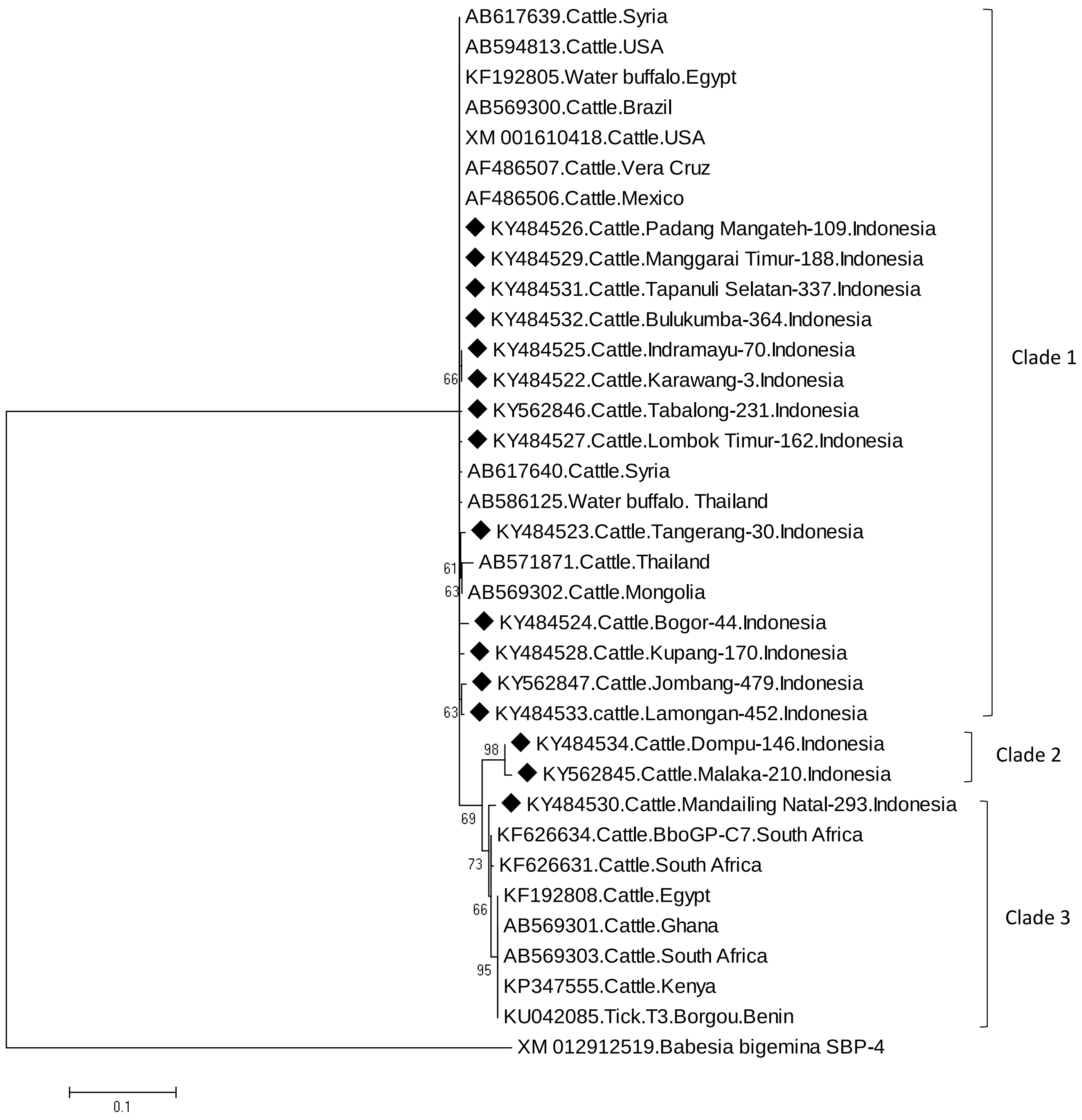

Additional file 4: Figure S2 Phylogenetic analysis of *Babesia bigemina* rap-1a gene sequences. The maximum likelihood method based on the Kimura 2-parameter model with 1000 bootstrap replicates, available in MEGA ver.7, was used to determine the evolutionary history [30, 40]. All positions containing gaps and missing data were eliminated. Indonesian *B. bigemina* rap-1a sequences are indicated by diamonds.

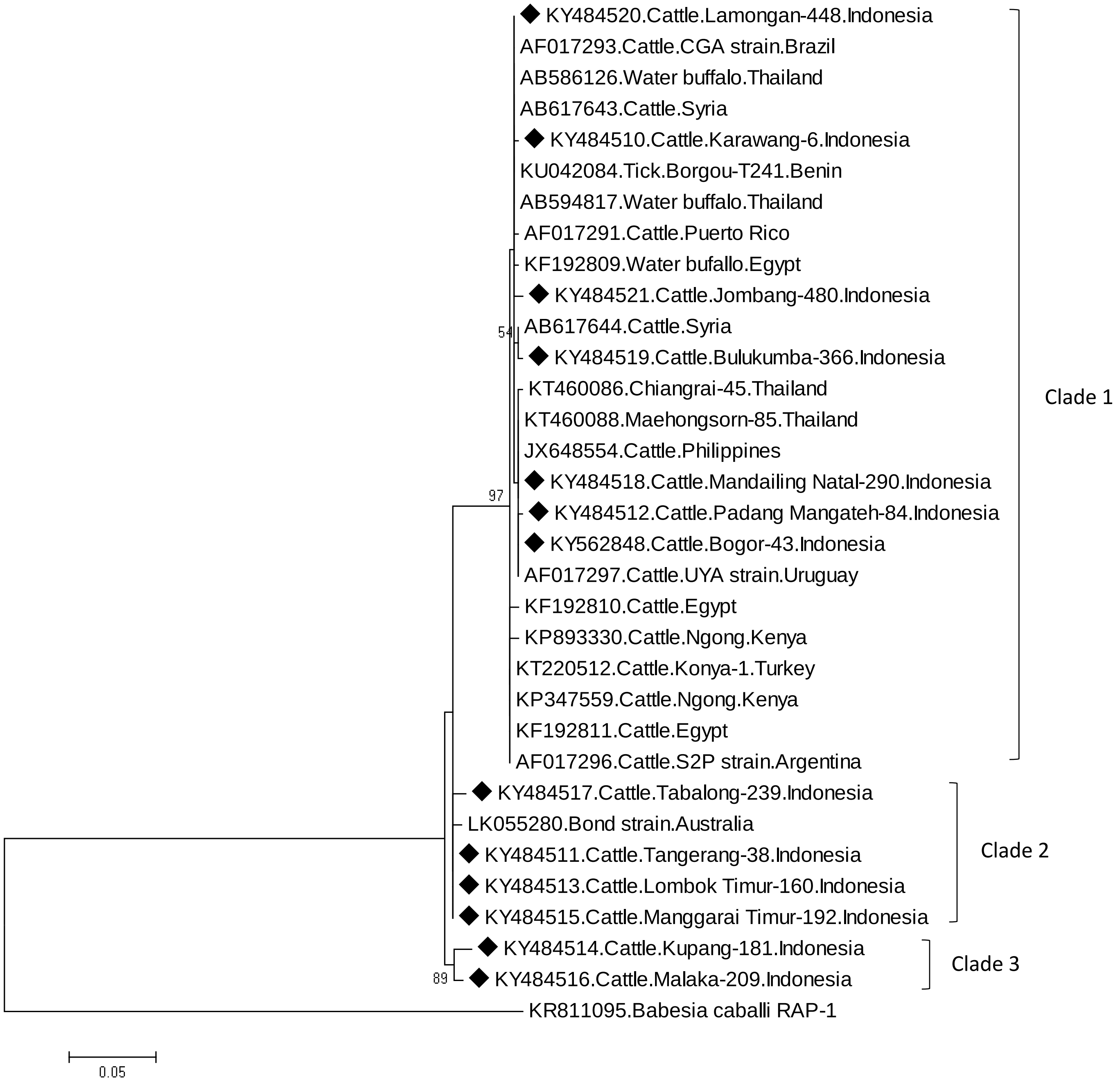

Supplement: Supplementary file 4 — Figure S1. Phylogenetic tree of Babesia bovis SBP-4 gene sequences. Figure. S2. Phylogenetic analysis of Babesia bigemina RAP-1a gene sequences. (PDF 170 kb) [file 13071_2017_2502_MOESM4_ESM.pdf]
